# Dual orexin receptor antagonism with lemborexant enhances microglial clearance of β-amyloid in mice

**DOI:** 10.1186/s13024-026-00948-y

**Published:** 2026-05-22

**Authors:** Ashish Sharma, Emiko Segawa, Xiaoying Chen, Sohui Park, Shoutang Wang, Riley E. Irmen, Nicholas J. Constantino, Chanung Wang, Michael F. Kanan, Marco Colonna, Shannon L. Macauley, Jocelyn Y. Cheng, Ken Hatanaka, Margaret Moline, Erik S. Musiek

**Affiliations:** 1 Department of Neurology, Hope Center for Neurological Disorders, Washington University School of Medicine, St. Louis, MO USA; 2 Eisai Co., Ltd., Tsukuba, Ibaraki Japan; 3 Laboratory of System Neuroimmunology, Institute for Immunology at Tsinghua, IDG/McGovern Institute for Brain Research at Tsinghua, School of Basic Medical Sciences, Tsinghua Medicine, Tsinghua University, Beijing, China; 4 Department of Pathology and Immunology, Washington University School of Medicine, St. Louis, MO USA; 5 School of Biomedical Sciences, Li Ka Shing Faculty of Medicine, The University of Hong Kong, Pok Fu Lam, Hong Kong, China; 6 Department of Physiology, Department of Neuroscience, Sanders Brown Center on Aging, University of Kentucky, Lexington, KY USA; 7 Eisai Inc., Nutley, NJ USA

**Keywords:** Alzheimer’s disease, Amyloid plaques, Sleep, Orexin, Microglia

## Abstract

**Background:**

Sleep disturbances elevate brain amyloid-beta (Aβ) levels and represent a modifiable risk factor for Alzheimer’s disease (AD). The orexin/hypocretin system regulates sleep–wake behavior and has emerged as a therapeutic target in AD; however, the effects of FDA-approved dual orexin receptor antagonists (DORAs) on amyloid pathology remain unclear. We compared lemborexant, an FDA-approved DORA, to doxepin, an antihistaminergic sleep medication, on amyloid pathology and microglial responses in PSAPP mice.

**Methods:**

PSAPP mice received lemborexant (10 or 30 mg/kg/day), doxepin (35 mg/kg/day), or vehicle for 6 weeks beginning prior to plaque onset or 4 weeks after established pathology. Sleep was assessed by piezoelectric monitoring and EEG/EMG polysomnography. Amyloid pathology and microglial responses were quantified by immunohistochemistry, confocal microscopy, and single-cell RNA sequencing. Microglial depletion was induced with the CSF1R inhibitor PLX3397.

**Results:**

Lemborexant enhanced sleep quality with less active-phase sedation than doxepin. Both drugs reduced initial diffuse plaque deposition, but only lemborexant prevented fibrillar plaque accumulation in young mice and slowed plaque growth in older mice. Lemborexant increased peri-plaque microglial CD68 expression and enhanced Aβ phagocytosis in vivo. Single-cell transcriptomics revealed a shift toward activated, DAM-like microglial states with upregulation of phagocytic genes without broad inflammatory induction. Microglial depletion abolished lemborexant’s anti-amyloid effects.

**Conclusions:**

Lemborexant mitigates amyloid pathology by augmenting microglial phagocytic function, positioning DORAs as promising therapeutics that couple sleep promotion with beneficial microglial modulation.

**Supplementary information:**

The online version contains supplementary material available at 10.1186/s13024-026-00948-y.

## Background

Alzheimer’s disease (AD) is a progressive neurodegenerative disorder characterized by amyloid-beta (Aβ) plaque deposition, tau neurofibrillary tangles, neuronal loss, and cognitive decline [1]. Among the modifiable factors that accelerate disease progression, sleep disturbance has emerged as a key contributor [2, 3]. Disrupted sleep elevates neuronal production and release of Aβ and tau [4–6], impairs glymphatic clearance of metabolic waste [7–9], and alters microglial reactivity in ways that promote neuroinflammation [10, 11].

The orexin/hypocretin system sits at the intersection of these mechanisms. As a master regulator of sleep–wake homeostasis [12, 13], the orexin system—acting through two G-protein-coupled receptors [14]—modulates both arousal and Aβ dynamics. Almorexant, an early dual orexin receptor antagonist (DORA), prevented plaque accumulation in APPswe/PS1δE9 (PSAPP) mice [4, 15], and three DORAs subsequently received FDA approval for insomnia treatment: suvorexant, lemborexant, and daridorexant [16, 17]. Suvorexant has also been shown to reduce soluble Aβ and phospho-tau in human cerebrospinal fluid [18]. Whether FDA-approved DORAs can directly reduce amyloid plaque burden, however, and the mechanisms by which they might do so, remain unknown.

To address this gap, we evaluated lemborexant (LEM), an FDA-approved DORA with low-nanomolar affinity for both orexin receptors and preferential OX2R binding [19], on amyloid pathology in PSAPP mice [20]. We compared LEM to doxepin (DOX), an FDA-approved antihistamine prescribed for insomnia [21], hypothesizing that LEM’s targeted orexin modulation would confer additional therapeutic benefit beyond improved sleep. Both drugs reduced total amyloid deposition early in disease, but only LEM prevented fibrillar plaque accumulation in young mice and slowed plaque growth in older animals. Mechanistically, LEM enhanced microglial Aβ phagocytosis in vivo and drove a transcriptional shift toward a reactive phenotype without broadly upregulating inflammatory cytokines. Together, these findings reveal a novel immunomodulatory role for orexin receptor antagonism in facilitating microglial-mediated Aβ clearance.

## Methods

### Ethics declarations

All animal procedures were reviewed and approved by the Institutional Animal Care and Use Committee (IACUC) of Washington University (Animal Welfare Assurance #D16-00245; protocol #23–0199) and conducted in strict accordance with the Guide for the Care and Use of Laboratory Animals (National Academies Press, 8th edition). Animals were maintained under specific pathogen-free conditions with a 12-h light/dark cycle, ambient temperatures of 20–26 °C, and 30–70% humidity, with ad libitum access to food and water. Animal care and veterinary oversight were provided by the Department of Comparative Biology.

### Study design

Male and female APPswe/PS1δE9 (PSAPP) mice were used for all experiments [20]. Because females develop plaques earlier, sexes were age-staggered in the younger cohort to initiate treatment just prior to plaque onset; both sexes began treatment simultaneously in older mice with established pathology. Mice were randomized by sex to experimental groups, and investigators were blinded during data acquisition and analysis. Group sizes were determined by power calculations based on pilot data to detect a 50% change in plaque burden with 80% power at α = 0.05, with sexes combined (not powered for sex-specific effects). Primary endpoints—plaque burden and peri-plaque microglial markers—were specified a priori; follow-up experiments were designed subsequently.

Mice received daily oral gavage at Zeitgeber Time 0 (ZT0; lights on) of vehicle (VEH; 0.5% methylcellulose in PBS), doxepin (DOX; 35 mg/kg; Cayman Chemical #15888, in PBS), or lemborexant (LEM; 10 or 30 mg/kg; provided by Eisai, in 0.5% methylcellulose PBS). For plaque-labeling and phagocytosis studies, Methoxy-X04 (MX-04; 10 mg/kg; Tocris #4920) was administered intraperitoneally.

**Fig. 1:**
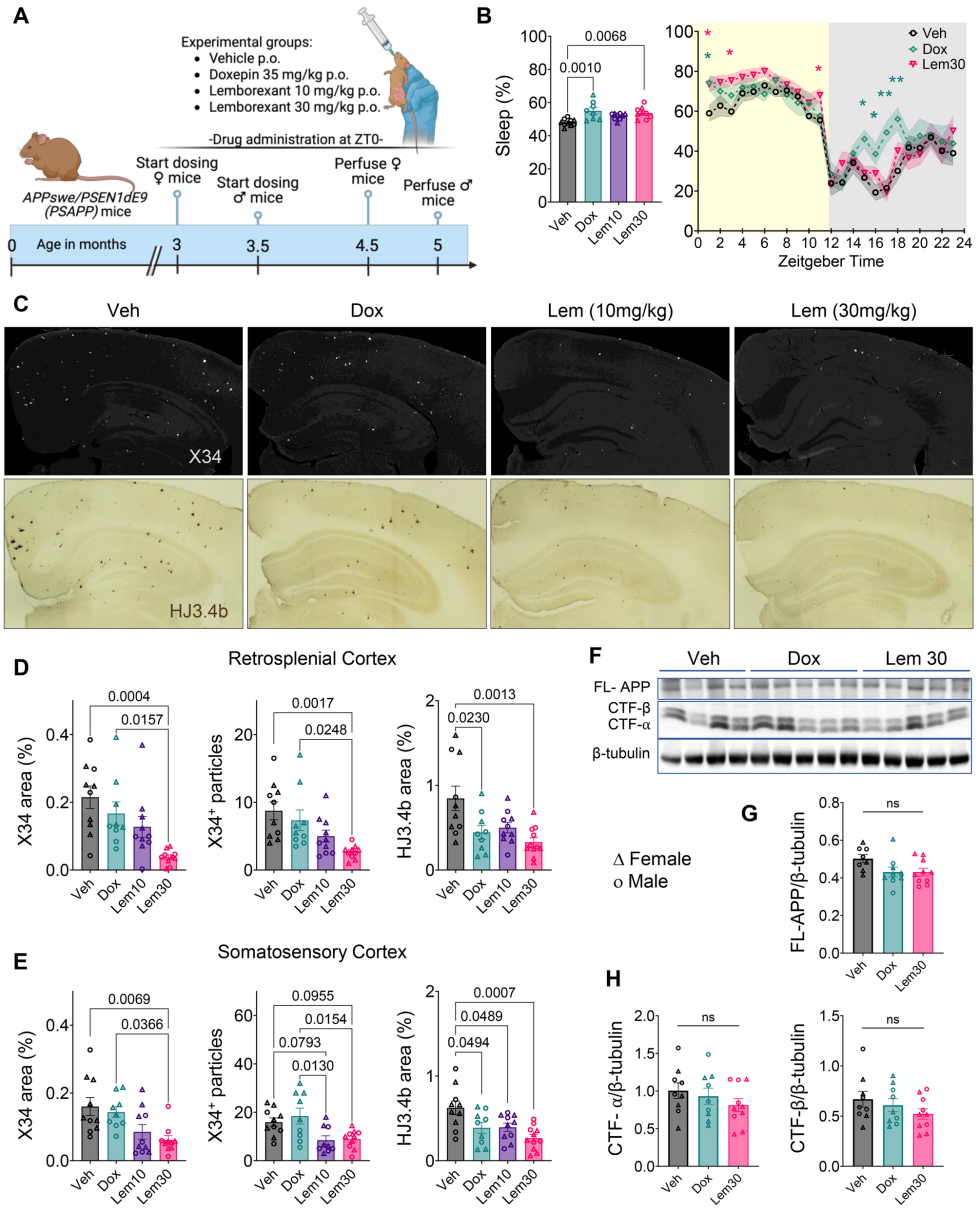
Lemborexant enhances sleep and suppresses de novo amyloid deposition in PSAPP mice. (**A**) Experimental timeline and dosing scheme. (**B**) Average total sleep over 24 h and across the circadian cycle for VEH, DOX (35 mg/kg), LEM10 (10 mg/kg), and LEM30 (30 mg/kg) groups; ZT0 = lights on. (**C–E**) Representative images and quantification of fibrillar plaques (X34) and total Aβ-immunopositive plaques (HJ3.4b) in retrosplenial and somatosensory cortices. (**F–H**) Immunoblots and densitometry of full-length APP (FL-APP) and C-terminal fragments (CTF-α, CTF-β); β-tubulin serves as loading control. Data are mean ± SEM; *p < 0.05, **p < 0.01, ***p < 0.001, ****p < 0.0001; exact *p* values shown in graphs

For de novo plaque deposition studies, three-month-old female and 3.5-month-old male PSAPP mice received VEH, LEM (10 or 30 mg/kg/day), or DOX (35 mg/kg/day) for six weeks. This age was chosen to initiate drug treatment prior to plaque appearance, and duration was chosen to allow for adequate plaque deposition. Microglial phagocytic activity was assessed in five-month-old mice, an age with mild plaque burden. Mice were treated with VEH or LEM (30 mg/kg/day) for seven days, followed by MX-04 injection and microglial isolation for flow cytometry. For plaque growth studies, nine-month-old mice, which have moderate plaque pathology, received MX-04 to label pre-existing plaques, then VEH, LEM (30 mg/kg/day), or DOX (35 mg/kg/day) for four weeks. Experimental timelines are shown in Fig. 1A, S1A, S2A, 3A, S6A, S6D, and 6A.

### Cell-free Aβ fibrillization assay

Synthetic Aβ1–42 peptide (Anaspec, AS-20276) was dissolved in 100% DMSO (Sigma, D2680) to make a 500 μM stock solution. The stock was diluted in PBS to a final concentration of 20 μM with or without 0.5, 1, or 2 μM of LEM, 0.5, 1, or 2 μM of orexin-A (OXA, Anaspec, AS-24470), or 10 μM of tannic acid (Sigma, 43040), in the presence of 20 µM Thioflavin-T (Sigma-Aldrich, 596200). Each sample was transferred to a clear-bottom 96 well pate in triplicates and incubated at 37 °C with constant shaking at 220 rpm. Fluorescence intensities at excitation = 485 nm/emission = 528 nm were recorded 1-hour intervals (0-9 h time points) using Gen5 software (v3.08) on BioTek Synergy HTX Multi-Mode Microplate Reader.

### Pexidartinib treatment

Pexidartinib (PLX3397; MedChemExpress Hy-16749) was formulated into AIN-76A rodent chow at 400 ppm (Research Diets, Inc.), irradiated, and provided ad libitum. To assess the role of microglia in LEM’s effects, PSAPP mice began PLX3397 diet at 8.5 months, then at 9 months received MX-04 for plaque labeling followed by VEH or LEM treatment for four weeks while continuing PLX3397 throughout.

### Piezoelectric sleep monitoring

To evaluate the effects of LEM and DOX on sleep–wake behavior, five-month-old PSAPP mice received daily treatments at ZT0 for six consecutive days with VEH, LEM (10 or 30 mg/kg/day), or DOX (35 mg/kg/day) (Supp. Fig. S1A). Sleep–wake states were continuously monitored using a noninvasive piezoelectric system [22] housed within a light- and sound-controlled circadian cabinet (Actimetrics, Inc.). Piezoelectric signals were analyzed using overlapping 8-second windows with 2-second epochs. For each window, a decision statistic was computed based on signal regularity (frequency and amplitude consistency) and peak spectral energy. Signals with greater regularity, peak energy in the breathing range (1–4 Hz), and lower relative amplitude yielded higher decision statistic values, indicating increased sleep probability. Linear discriminant analysis classified sleep–wake states from extracted signal features [22]. This method distinguishes wakefulness from sleep with 89% sensitivity and 96% specificity [23]. All experiments were conducted under 12:12-hour light–dark conditions.

### EEG/EMG polysomnography

Sleep architecture was assessed by EEG/EMG in a subset of five-month-old PSAPP mice. Stereotaxic surgery was performed as previously described [24, 25]. Briefly, mice were anesthetized with 5% isoflurane and maintained at 1.5–2.0% isoflurane during surgery under sterile conditions. Two stainless steel bone screws (MD-1310, BASi Research Products) serving as EEG leads were placed over the right frontal cortex (A/*P* +1 mm, M/L −1 mm) and left parietal cortex (A/*P* −2 mm, M/L +1 mm), with a reference screw at the cerebellum (A/*P* −6 mm, M/L 0 mm). Insulated wire leads soldered to a headmount (8402, Pinnacle Technology) were wrapped around the bone screws. Stainless steel wires attached to the headmount were inserted into neck musculature for EMG recording. Screws, wires, and headmount were secured with dental acrylic. Mice recovered for 5 days in recording cages (Pinnacle Technology) under 12:12-hour light–dark conditions before recording.

Raw EEG data were analyzed using Sirenia Sleep Pro and manually scored in 10-second epochs according to standard wake, NREM, and REM classifications [24, 25]. Percent time per hour in each state was calculated across the 24-hour day. Mice received VEH, LEM (30 mg/kg/day), and DOX (35 mg/kg/day) at ZT0 for 3 consecutive days each, with a 3-day washout between LEM and DOX treatments (Supp Fig. S2A).

### Tissue collection

To minimize circadian variability, all mice were perfused between ZT5 and ZT7. Mice were deeply anesthetized with pentobarbital (150 mg/kg, i.p.) and transcardially perfused with ice-cold Dulbecco’s PBS (DPBS) containing 3 g/L heparin. The cortex and hippocampus from one hemibrain were dissected, snap-frozen on dry ice, and stored at − 80 °C. The contralateral hemibrain was immersion-fixed in 4% paraformaldehyde for 24 h at 4 °C, cryoprotected in 30% sucrose for 48 h, and stored at − 20 °C until sectioning. Coronal brain sections (40 µm) were obtained using a freezing sliding microtome (SM1020R; Leica) and stored in cryoprotectant solution for subsequent immunohistochemical or immunofluorescence analyses.

### Immunohistochemistry

Free-floating coronal sections (2 per mouse) were washed in TBS, quenched with 0.3% hydrogen peroxide for 10 min, then blocked with 3% milk in 0.25% TBS-X (TBS + Triton X-100) for 30 min at room temperature. Sections were incubated overnight at 4 °C with biotinylated HJ3.4 antibody (mouse monoclonal, D. Holtzman laboratory), which recognizes the N-terminus of human Aβ (residues 1–16) and detects total Aβ including diffuse and dense-core plaques [26]. Sections were then washed, incubated with ABC Elite reagent (Vector Laboratories, PK-6100) for 1 h, and developed with 3,3′-diaminobenzidine (DAB; Sigma-Aldrich) for 10 min. Sections were mounted, dehydrated through graded ethanol, cleared in xylene, and coverslipped with Cytoseal. Slides were imaged at 10× using brightfield microscopy (Keyence BZ-X).

### Immunofluorescence

Dense core fibrillar plaques were stained with X34 or Thiazine Red. For X34, free-floating sections were washed in PBS, permeabilized in 0.4% PBS-X for 30 min, then incubated in X34 staining buffer (10 µM X34 [Sigma-Aldrich SML1954] in 60% PBS/40% ethanol with 0.02 N NaOH) for 20 min. Sections were rinsed in 60% PBS/40% ethanol then PBS, blocked for 60 min in 0.4% TBS-X with 5% donkey serum, and incubated overnight at 4 °C with primary antibodies in TBS-X + 1% donkey serum. Sections were then washed, incubated with fluorescent secondary antibodies (1:1,000 in TBS-X) for 1 h at room temperature, and mounted with Fluoromount-G (SouthernBiotech 0100–01). For Thiazine Red, sections were washed in TBS, permeabilized in 0.25% TBS-X for 30 min, incubated in 2 µM Thiazine Red (Chemsavers THIR1G) for 20 min, washed three times for 5 min in TBS, and mounted.

Primary antibodies: IBA1 (rabbit, Wako 019–19741, 1:1,000; or goat, Abcam ab5076, 1:500), GFAP-Alexa Fluor 647 (mouse, Cell Signaling 3657S, 1:800), CD68 (rat FA-11, Bio-Rad MCA1957, 1:1,000), APOE (mouse HJ6.3, D. Holtzman laboratory, 1:1,000), P2RY12 (rat S16007D, BioLegend 848002, 1:100). Secondary antibodies (all 1:1,000, Invitrogen): donkey anti-mouse IgG–Alexa Fluor 488 (A-21202), donkey anti-rat IgG–Alexa Fluor 488 (A-21208), donkey anti-rabbit IgG–Alexa Fluor 568 (A-10042), donkey anti-goat IgG–Alexa Fluor 568 (A-11057), donkey anti-goat IgG–Alexa Fluor 647 (A-21447), donkey anti-rabbit IgG–Alexa Fluor 647 (A-31573), donkey anti-rat IgG–Alexa Fluor 647 (A-78947).

### Imaging acquisition and analysis

Epifluorescent images were acquired on a Keyence BZ-X810 microscope. Laser intensity and exposure times were optimized using representative sections and held constant across all samples within each cohort. Whole-section mosaics were stitched with BZ-X800 Analyzer (Keyence) and quantified in Fiji v2.16.0 (NIH). For peri-plaque analyses, cortical fibrillar plaques were imaged on a Zeiss LSM-980 Airyscan 2 confocal microscope with ZEN 3.6 (blue edition); laser power, detector gain, and pinhole size were held constant within each experiment. At least 10 cortical plaques per mouse were captured at 20× or 40× (1,024 × 1,024 pixels, 0.3 µm z-steps) with Airyscan adaptive deconvolution for super-resolution. Images were quantified in MATLAB and Imaris 10.0.1 (Bitplane); a 20 µm three-dimensional shell was projected around each X34-positive plaque, with all fluorescence within this volume considered peri-plaque signal.

### Protein extraction

Frozen cortical tissue was weighed and homogenized in a Bullet Blender (Next Advance) with 250 µL RIPA buffer (Pierce, Thermo Scientific) supplemented with protease inhibitor (cOmplete; Roche) and phosphatase inhibitor (PhosSTOP; Roche). Homogenates were centrifuged at 5,000 g for 5 min at 4 °C and supernatants collected. Protein concentrations were determined in duplicate by BCA assay (Thermo Scientific). Aliquots were stored at − 80 °C until analysis.

### Immunoblotting

Protein (10 µg) was mixed with NuPAGE reducing agent (Invitrogen) and 4× Laemmli sample buffer, then resolved on Novex 16% Tricine gels (Invitrogen) in Tricine SDS running buffer with NuPAGE antioxidant. Proteins were transferred to PVDF membranes and blocked in 5% milk/TBST for 60 min. Membranes were incubated overnight at 4 °C with primary antibodies: β-amyloid (rabbit CT695, Invitrogen 51-2700, 1:1,000) and β-tubulin (mouse IgG2a, Invitrogen MA5-16308, 1:2,000). After washing, HRP-conjugated secondary antibodies were applied for 1 h. Signal was developed with Pierce ECL or Lumigen ECL Ultra and imaged on a Bio-Rad ChemiDoc system. Band intensities were quantified in Fiji and normalized to β-tubulin. For sequential probing, membranes were stripped with Restore PLUS buffer (Thermo Scientific) at 37 °C for 30 min with agitation, rinsed in distilled water, washed 3 × 10 min in TBST, re-blocked, and reprobed.

### *In vitro* microglial uptake and degradation assays

BV2 cells were cultured in DMEM (Gibco 11965-092) with 10% FBS (Biowest S1620) and 100 U/mL penicillin-streptomycin (Gibco 15140-122). For uptake and degradation assays, cells were seeded at 50,000 cells/well in 24-well plates and incubated overnight. Cells were pretreated for 30 min with 200 µM Dynasore (Sigma D7693), 10 µM cytochalasin D (Cayman Chemical 11330), or orexin-A (OXA; 0.5, 1, or 2 µM). Media was replaced with fresh media containing the respective inhibitor or OXA plus fluorescently labeled substrates: 1 µg/mL fibrillar Aβ-488 (Anaspec AS-60479-01), Alexa Fluor 647-BSA (Invitrogen A34785), or DQ-BSA (Invitrogen D12051). After 1 h, cells were washed twice with DPBS (Gibco 14190-144), trypsinized (0.05% trypsin-EDTA; Gibco 25300-054), and resuspended in flow cytometry buffer (DPBS with 1% FBS and 1 mM EDTA). Flow cytometry was performed on a Beckman Coulter CytoFLEX S and analyzed in FlowJo v10.10.0 with gating for single, live cells.

For LEM experiments, cells were treated for 1 h with 200 µM Dynasore, 20 µM cytochalasin D, or LEM (1 or 10 µM), then media was replaced with the respective treatment plus fibrillar Aβ-488, 647-BSA, or DQ-BSA. A subset of LEM-treated cells also received 1 µM orexin-A. Cells were incubated for 1 h and collected for flow cytometry.

### Single‑cell suspension preparation

All steps were performed on ice or at 4 °C. Mice were transcardially perfused with pre-chilled DPBS. Cortex and hippocampus were dissected and mechanically dissociated using a glass Dounce homogenizer [27]. Homogenates were subjected to Percoll density centrifugation to deplete myelin and debris, and cell pellets were rinsed in 0.5% BSA/DPBS for downstream assays.

### *In vivo* microglial Aβ phagocytosis assay

In vivo microglial Aβ phagocytosis was assessed as described [28, 29]. Single-cell suspensions were pre-incubated for 5 min on ice with anti-CD16/32 Fc block (eBioscience 14–0161-85, 1:100), which remained present throughout staining. Cells were labeled on ice for 20 min with CD45-PE (clone 30-F11, BioLegend, 1:100) and CD11b-APC (clone M1/70, BioLegend, 1:100), washed, and resuspended in 5% BSA. Samples were acquired on an LSR Fortessa (BD Biosciences) and analyzed in FlowJo. Microglia were defined as CD45^lo^CD11b^+^, and the percentage of MX-04-positive cells was quantified.

### Fluorescence activated cell sorting

PSAPP mice (*n* = 5 per group) received daily VEH or LEM (30 mg/kg/day) by oral gavage for one month beginning at 9 months of age. At 10 months, mice were perfused; one hemisphere was fixed for immunofluorescence, and cortex and hippocampus from the contralateral hemisphere were processed into single-cell suspensions. Following Fc block with anti-CD16/32, cells were stained with CD45-FITC and CD11b-PE for 20 min on ice. After washing, DAPI was added for viability, and live microglia (CD45^lo^CD11b^+^ DAPI^−^) were sorted using a FACSAria II (BD Biosciences) into 1% BSA/PBS. Sorted cells were submitted to the institutional Genomics Core for library preparation and single-cell RNA sequencing.

### scRNA-seq and read alignment

After confirming cell viability and integrity, approximately 20,000–30,000 single cells per sample were loaded onto a 10X Genomics Chromium platform to generate gel beads-in-emulsion (GEMs). GEMs were used to synthesize cDNA carrying cell-specific and transcript-specific barcodes. Sequencing libraries were prepared using the 10X Genomics Chromium Single Cell 5’ PE Library & Gel Bead Kit V2 and sequenced on an Illumina NovaSeq6000. Sequencing data were processed using Cell Ranger (v8.0.0) [30] for alignment, barcode assignment, and UMI counting, generating gene-expression count matrices mapped to the mm10-2020-A mouse transcriptome.

### Data preprocessing and quality control

All data processing and statistical analyses with scRNA-seq data were conducted using R (v4.4.0). Firstly, ambient RNA contamination was removed, followed by the selection of high-quality cells with sufficient sequencing depth. Cell Ranger-filtered HDF5 expression matrices containing cells expressing fewer than 200 genes and genes expressed in fewer than 3 cells were subjected to SoupX (v1.6.2) [31], with default parameters. Subsequently, additional filtering was performed to remove cells expressing fewer than 500 genes, with more than 20,000 total counts, or with mitochondrial RNA content exceeding 2.5%. High-quality cells retained after filtering were used for downstream analysis.

### Data normalization, dimensional reduction, and clustering

Preprocessed data were imported into Seurat (v5.3.0) [32] to be normalized, scaled and centered before proceeding to clustering and microglia subsetting. Log-normalization was applied, and highly variable genes were identified using variance-stabilizing transformation. Dimensionality reduction was performed using principal component analysis (PCA) with 2000 variable genes, followed by Uniform Manifold Approximation and Projection (UMAP). Clustering was conducted using the FindNeighbors and FindClusters functions with k.param = 30 and resolution = 1.0.

### Cell type annotation and microglia subsetting

Cell types were manually annotated based on canonical marker genes, and microglia clusters were selected for downstream analysis followed by re-scaling and re-clustering. Microglia were identified using *Ctss, C1qa, C1qb, Cx3cr1, Hexb*, and *P2ry12*. Neuronal cells were classified using *Grin1, Syt1, Rbfox3, Gad1, Gad2*, and *Sst*. Astrocytes were identified using *Aqp4, Gfap*, and *Slc1a2*; oligodendrocytes using *Mog, Mag*, and *Olig2*; oligodendrocyte precursor cells (OPCs) using *Pdgfra, Vcan*, and *Olig1*; endothelial cells using *Pecam1, Cdh5,* and *Cldn5*; and pericytes using PDGFRB. To classify T cells, B cells, natural killer cells, or neutrophils, markers genes for distinct brain BAM subpopulations [33] were used.

Clusters identified as non-microglial clusters based on low microglial marker expression and high expression of other cell-type markers were excluded from further analysis. The resulting clusters were re-scaled and re-clustered using FindNeighbors and FindClusters functions with k.param = 30 and resolution = 0.9, based on the top principal components determined by elbow plot inspection. UMAP was then computed using the top 20 PCs. Homeostatic, interferon-responsive, Proliferating, DAM1, and DAM2 marker genes [34] were used to annotate microglial subtypes after all data processing.

### Statistical analyses

To identify clusters with proportional changes, permutation analysis was performed with 1,000 permutations. Clusters with FDR < 0.05 and log2 fold-change(FC) >0.58 were considered significant. Differential expression analysis was conducted using FindAllMarkers and FindMarkers functions, applying the Wilcoxon rank-sum test with false discovery rate (FDR) correction between clusters or LEM and VEH condition. Genes expressed in ≥10% of cells within a group and with a log2 FC > 0.1 were considered differentially expressed. A Curated set of well-established genes associated with microglial activation states and functional phenotypes were visualized. Enrichment analysis was performed using KEGG Database (Release 115.1, August 1, 2025) [35–37] and clusterProfiler (v4.14.6) [38], applying hypergeometric testing and FDR correction. For visualization, adjusted *p*-values were transformed using −log₁₀. For each differentially proportional cluster, all FDR-significant pathways were compiled (see Supplementary File). From the ten pathways with the lowest FDR per cluster, those most relevant to neurodegeneration or microglial function—as well as those recurring across multiple clusters—are highlighted in Fig. 4E.

All analyses except scRNA-seq were performed in GraphPad Prism v10.5.0. Data are presented as mean ± SEM. Sample sizes were determined by power analysis. Outliers were identified using the ROUT method (Q = 1%). Group differences were assessed by one-way ANOVA with Tukey or Tukey–Kramer post-hoc tests as appropriate. For two-group comparisons, variance homogeneity was assessed by F-test; unpaired two-tailed t-tests were used for equal variances and Mann–Whitney U tests for unequal variances. Significance was defined as *p* < 0.05 (*p < 0.05, **p < 0.01, ***p < 0.001, ****p < 0.0005). Multiple comparison corrections were applied where appropriate; exact *p* values are reported in figures.

## Results

### Lemborexant enhances sleep quality without active-phase sedation

Sleep was recorded continuously for one week using a noninvasive PiezoSleep system that estimates sleep-wake states from movement and breathing frequency over 2-second epochs [22]. Five-month-old PSAPP mice with mild plaque pathology received daily oral gavage at ZT0 (lights on) of VEH, DOX (35 mg/kg), LEM10 (10 mg/kg), or LEM30 (30 mg/kg) for seven days (Supp. Fig. S1A).

We selected a higher DOX dose than previously published [39] to match the total sleep increase of LEM30 by piezoelectric measurement. Both DOX and LEM30 increased total sleep by approximately 8%, whereas LEM10 had no significant effect. However, LEM30-induced sleep enhancement was confined to the light (inactive) phase, while DOX augmented sleep across both light and dark periods (Fig. 1B and Supp. Fig. S1B,C). Although average 24-hour sleep and wake bout lengths did not differ between treatments, binned analysis revealed that LEM30 maintained shorter wake bouts during the light phase, indicating more consolidated rest (Supp. Fig. S1B,C). Thus, while DOX and LEM30 provide similar total sleep, only LEM30 preserves active-phase wakefulness—a therapeutically advantageous profile.

To validate these findings, sleep architecture was assessed in a subset of PSAPP mice using EEG/EMG. Mice sequentially received vehicle, LEM30, a washout period, then DOX. The piezoelectric system overestimated sleep compared to EEG/EMG, particularly in DOX-treated mice, particularly during the active (dark) phase (Supp Fig. S2B). By EEG/EMG, LEM30 significantly increased total and non-REM sleep across the rest phase, whereas DOX increased total sleep immediately after dosing and again mid-active phase without affecting REM or NREM (Supp Fig. S2C,D). This discrepancy suggests DOX increases quiet wakefulness during the active phase rather than inducing true sleep, consistent with its antihistamine sedative profile.

### Lemborexant selectively prevents fibrillar plaque accumulation

To assess plaque prevention, PSAPP mice received daily treatment at ZT0 for six weeks with VEH, DOX, LEM10, or LEM30 (Fig. 1A), beginning just before plaque onset. Females started at three months and males at 3.5 months to accommodate sex-dependent plaque kinetics. At 4.5 (females) or 5 (males) months, brains were perfused and processed for immunohistochemistry. We focused on retrosplenial, somatosensory, and piriform cortices—regions with early plaque deposition in PSAPP mice—using hippocampus as an internal comparator given its delayed plaque accumulation. X34 staining revealed significant reductions in fibrillar, dense-core plaque number and area in retrosplenial and somatosensory cortices of LEM30-treated mice; DOX and LEM10 had no effect.

Using HJ3.4b—which detects both fibrillar and diffuse Aβ—to quantify total plaque burden, both DOX and LEM30 reduced plaques in retrosplenial cortex, and all treatments decreased plaques in somatosensory cortex relative to VEH (Fig. 1C–E). LEM10 and LEM30, but not DOX, also reduced plaques in piriform cortex, with no significant changes in hippocampus where minimal accumulation occurs at this age (Supp Fig. S3). Western blot analysis of cortical lysates showed unchanged APP and C-terminal fragment (CTF-α/β) levels in DOX- or LEM30-treated mice, indicating no effect on APP processing (Fig. 1F–H). Cell-free Aβ aggregation assays confirmed that neither LEM nor orexin-A directly alters fibrillization in vitro (Supp Fig. S4). Thus, while both sleep-promoting drugs reduce diffuse plaques, only LEM30 prevents maturation of fibrillar pathology in regions most vulnerable to early deposition.

### Lemborexant promotes microglial Aβ uptake

**Fig. 2:**
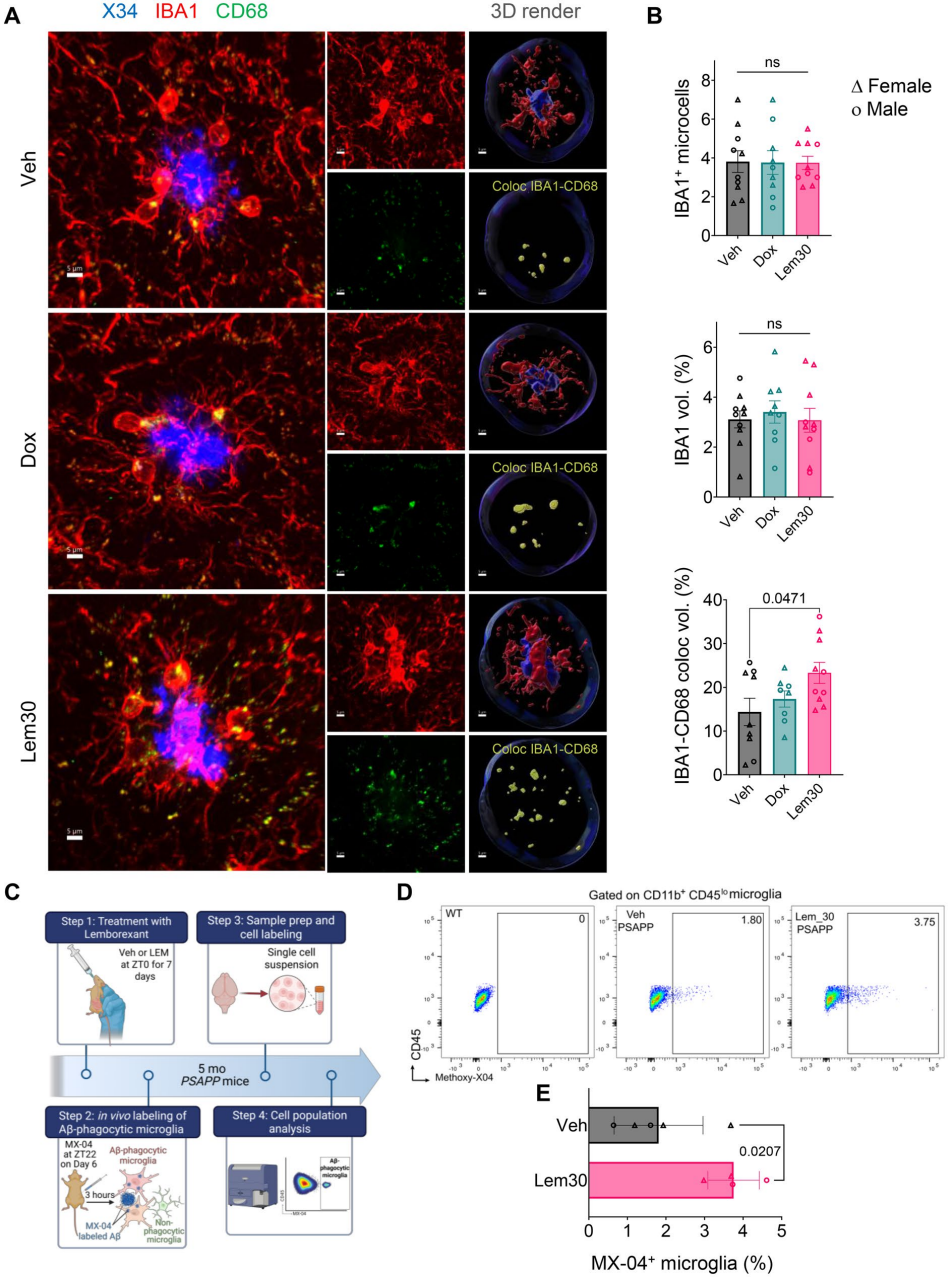
Lemborexant enhances microglial amyloid phagocytosis. (**A**) Representative 3D confocal renderings of peri-plaque microglia (IBA1) surrounding fibrillar plaques (X34), with colocalized phagosomal marker CD68. (**B**) Quantification of peri-plaque IBA1–CD68 colocalization in VEH, DOX, and LEM30 groups. (**C**) Schematic of in vivo Aβ phagocytosis assay. (**D–E**) Representative flow cytometry scatterplots and quantification of MX-04+ microglia (CD11b^+^CD45^lo^) after LEM30 treatment. Data are mean ± SEM; exact *p* values shown in graphs

To explore mechanisms underlying reduced amyloid accumulation with LEM, we first assessed glial activation. Total IBA1 immunoreactivity—a pan-microglial marker—remained unchanged across treatments in multiple brain regions (Supp Fig. S5A,B), as did GFAP, a marker for reactive astrocytes (Supp Fig. S5C,D). Since peri-plaque microglia adopt a phagocytic phenotype marked by CD68 (macrosialin) upregulation [40–43], we quantified IBA1+ microglia and CD68 expression within 20 µm of X34+ plaques using confocal microscopy and 3D Imaris reconstruction (Fig. 2A). The number and normalized volume of peri-plaque IBA1+ microglia were unchanged. However, LEM30, but not DOX, increased peri-plaque microglial CD68 expression relative to VEH (Fig. 2B), indicating enhanced phagocytic activation.

To determine whether elevated microglial CD68 translates to enhanced Aβ uptake, we labeled plaques in vivo with methoxy-X04 (MX-04) and quantified MX-04+ microglia by flow cytometry as an index of amyloid phagocytosis [28, 29]. Five-month-old PSAPP mice, which have mild plaque burden, received VEH or LEM30 at ZT0 for seven days, then MX-04 on day 7. Microglia were isolated three hours later and CD11b^+^CD45^lo^MX-04^+^ cells quantified (Fig. 2C). MX-04+ microglia were undetectable in wild-type controls. Vehicle-treated PSAPP mice exhibited ~2% MX-04+ microglia, whereas LEM30 nearly doubled this fraction to ~3.9% (Fig. 2D,E), demonstrating increased in vivo Aβ uptake.

We next tested whether LEM or orexin-A (OXA) directly affects microglial phagocytosis using BV2 cells (Supp Fig. S6). Cells were treated with OXA at various concentrations, and uptake of fluorescently labeled substrates (Aβ42–488, Alexa Fluor 647-BSA, or DQ-BSA) was measured by flow cytometry (Supp Fig. S6A); Dynasore and cytochalasin D served as negative controls. OXA had no effect on protein uptake or degradation at any concentration (Supp Fig. S6C). Similarly, LEM (1 or 10 µM), alone or with 1 µM OXA (Supp Fig. S6D), did not alter phagocytosis or degradation (Supp Fig. S6F). These results indicate that LEM does not directly modulate microglial phagocytosis but likely acts through indirect mechanisms.

### Lemborexant modulates amyloid pathology and microglial transcriptional states in advanced pathology

**Fig. 3:**
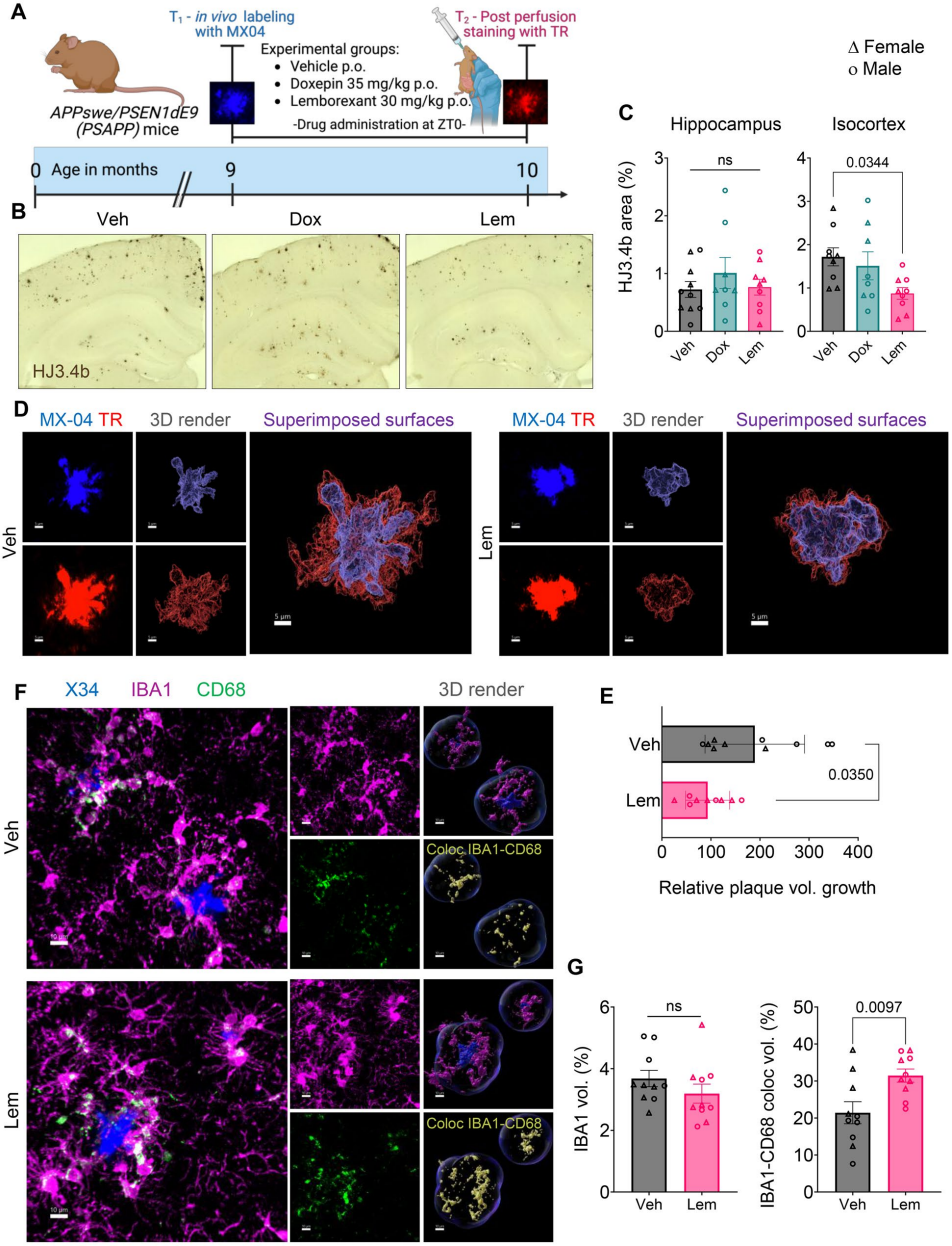
Lemborexant slows growth of established amyloid plaques. (**A**) Experimental timeline and dosing scheme. (**B–C**) Representative images and quantification of total plaque burden (HJ3.4b). (**D–E**) Representative MX-04/thiazine red 3D reconstructions and quantification of plaque core growth. (**F–G**) Representative images and quantification of peri-plaque IBA1–CD68 colocalization after LEM30 treatment. Data are mean ± SEM; exact *p* values shown in graphs

We next evaluated LEM30 (hereafter LEM) and DOX in 9-month-old PSAPP mice with established plaque pathology. To track growth of existing plaques, we “timestamped” plaques in vivo with i.p. MX-04 at 9 months. Mice then received daily VEH, DOX, or LEM at ZT0 for one month before perfusion and thiazine red re-labeling at 10 months (Fig. 3A). Total plaque burden by HJ3.4b IHC was significantly reduced in LEM- but not DOX-treated mice (Fig. 3B,C), though fibrillar plaque burden by thiazine red did not differ significantly (Supp Fig. S7). Given the lack of DOX effect, subsequent analyses focused on VEH vs. LEM. Confocal imaging and 3D reconstruction enabled precise measurement of plaque growth by subtracting MX-04+ from thiazine red+ volumes; LEM treatment slowed plaque growth by ~50% (Fig. 3D,E). Peri-plaque microglia in LEM-treated mice also exhibited increased CD68 expression, consistent with our findings in younger mice (Fig. 3F,G).

**Fig. 4:**
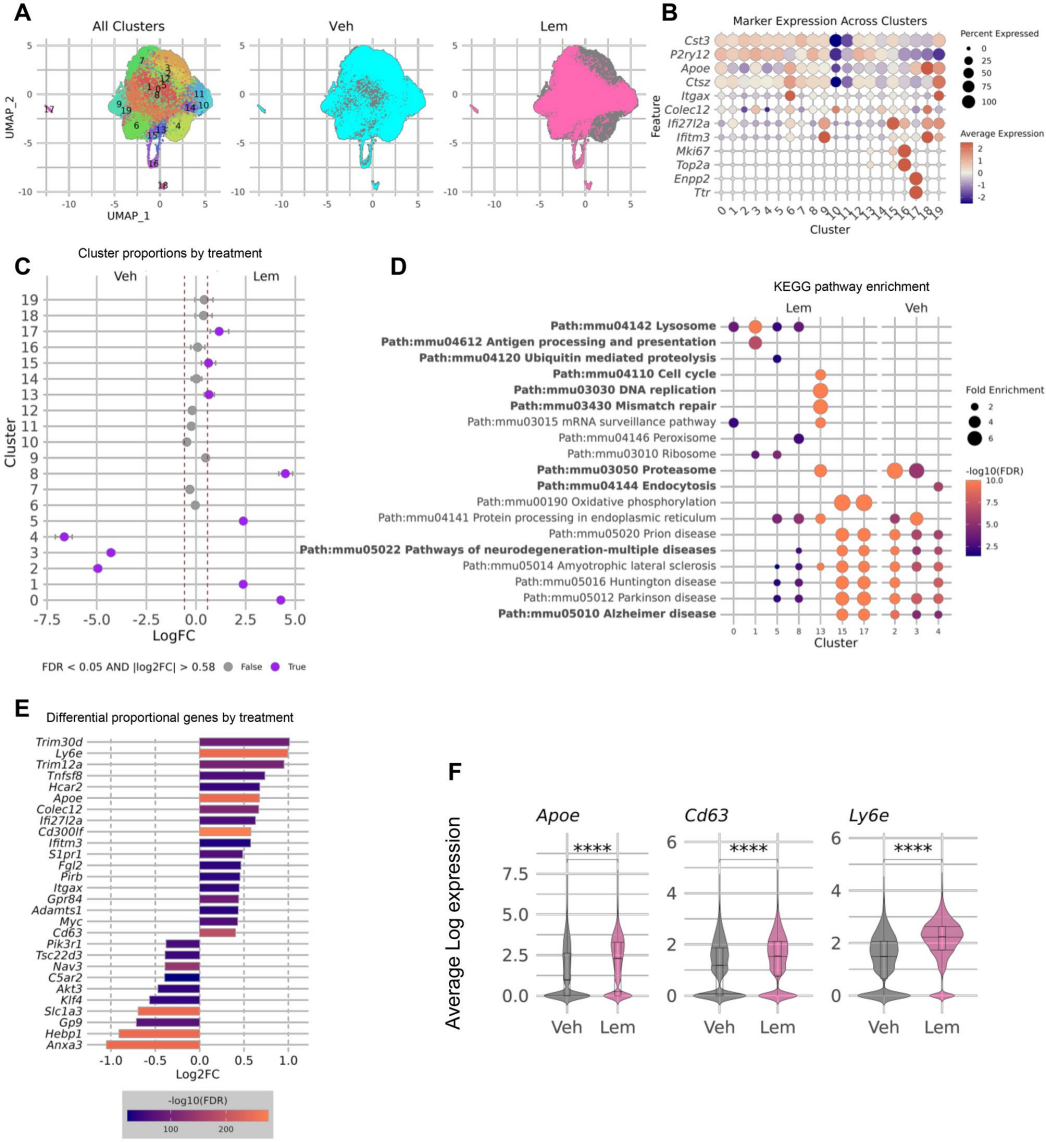
Single-cell transcriptomics reveal lemborexant-induced microglial state transitions. (**A**) UMAP visualization of 43,161 microglia resolved into 20 transcriptionally distinct clusters. (**B**) Expression of homeostatic, DAM, CD11c^+^, IFN-inducible, proliferative, and border-associated macrophage-like markers across clusters. (**C**) Cluster proportions by treatment; clusters 2, 3, 4 enriched in VEH; clusters 0, 1, 5, 8, 13, 15, 17 enriched in LEM. (**D**) Dot plot of select overrepresented KEGG pathways for VEH- or LEM-enriched clusters. (**E**) Select differentially proportional genes between VEH and LEM total microglial populations. **(F**) Log-average expression of select genes; *p < 0.0005 by Wilcoxon rank-sum test with FDR correction

To characterize LEM-induced microglial changes, we profiled 43,161 cortical and hippocampal microglia from VEH- or LEM-treated PSAPP mice (9–10 months; five pooled per group) by scRNA-seq. Unsupervised analysis resolved 20 transcriptionally distinct clusters, including disease-associated microglia (DAM; clusters 6, 19), interferon-responsive microglia (cluster 9), proliferative populations (clusters 13, 15, 16), and border-associated macrophage-like cells (cluster 17); homeostatic markers were broadly expressed in clusters 0–5, 7–9, and 12–15 (Fig. 4A,B).

Cluster-proportion analysis revealed no significant treatment effect on terminal DAM or IFN clusters, whereas homeostatic-like clusters 2, 3, and 4 were enriched in VEH-treated mice; markers from these clusters were over-represented in KEGG pathways linked to neurodegeneration, including AD (Fig. 4C,D). LEM markedly expanded clusters transcriptionally positioned between homeostatic and DAM states (clusters 0, 1, 5, 8), co-expressing both marker sets. Although canonical DAM gene upregulation in these clusters was modest, it was consistent and coupled to strong enrichment of antigen processing/presentation and lysosomal pathways (Fig. 4B–D), indicating a concerted shift toward an activated, phagocytic phenotype. LEM also increased proliferative microglia (clusters 13, 15) and border-associated macrophage-like cells (cluster 17) (Fig. 4C).

Differential proportional analysis across clusters showed that LEM increased the fraction of microglia expressing DAM markers (*Apoe, Cd63, Gpr84*) while elevating average expression of additional DAM-related transcripts (*Cd68, Ctsz, Axl, B2m, Ctsd, Lyz2, Tyrobp*) (Fig. 4E,F; Supp Fig. S8A). Conversely, the proportion expressing homeostatic markers (*C5ar2, Gp9, Slc1a3*) declined, and several classical homeostatic genes (*Cst3, Jun, P2ry12, Bin1, Cx3cr1, Serinc3, Tgfbr1, Tmsb4x*) showed reduced average expression without proportional changes (Fig. 4E; Supp Fig. S9) [44, 45]. The complete differentially proportional and expressed gene list is provided in the Supplementary File. LEM also increased both proportion and average expression of genes associated with phagocytosis (*Cd300lf, Colec12, Cd63, Gpr84, Fgl2, Hcar2, S1pr1*) and interferon responses (*Ifi27l2a, Ifitm3, Ly6e*), whereas MHC-I markers (*H2–D1, H2–K1, Pilra, Pirb*) increased only in average expression level (Fig. 4E; Supp Fig. S8). LEM elevated the CD11c+ population marked by *Itgax* and *Colec12* (Fig. 4E; Supp Fig. S8F), previously linked to developmental myelinogenesis and lysosome-rich phagocytes [46–48]. Cytokine and chemokine transcripts remained largely stable, with modest increases in *Il1a, Il1b, Il6, Il18*, and *Ccl5* and decreases in *Il15, Tnf*, and *Ccl12* (Supp Table T1).

**Fig. 5:**
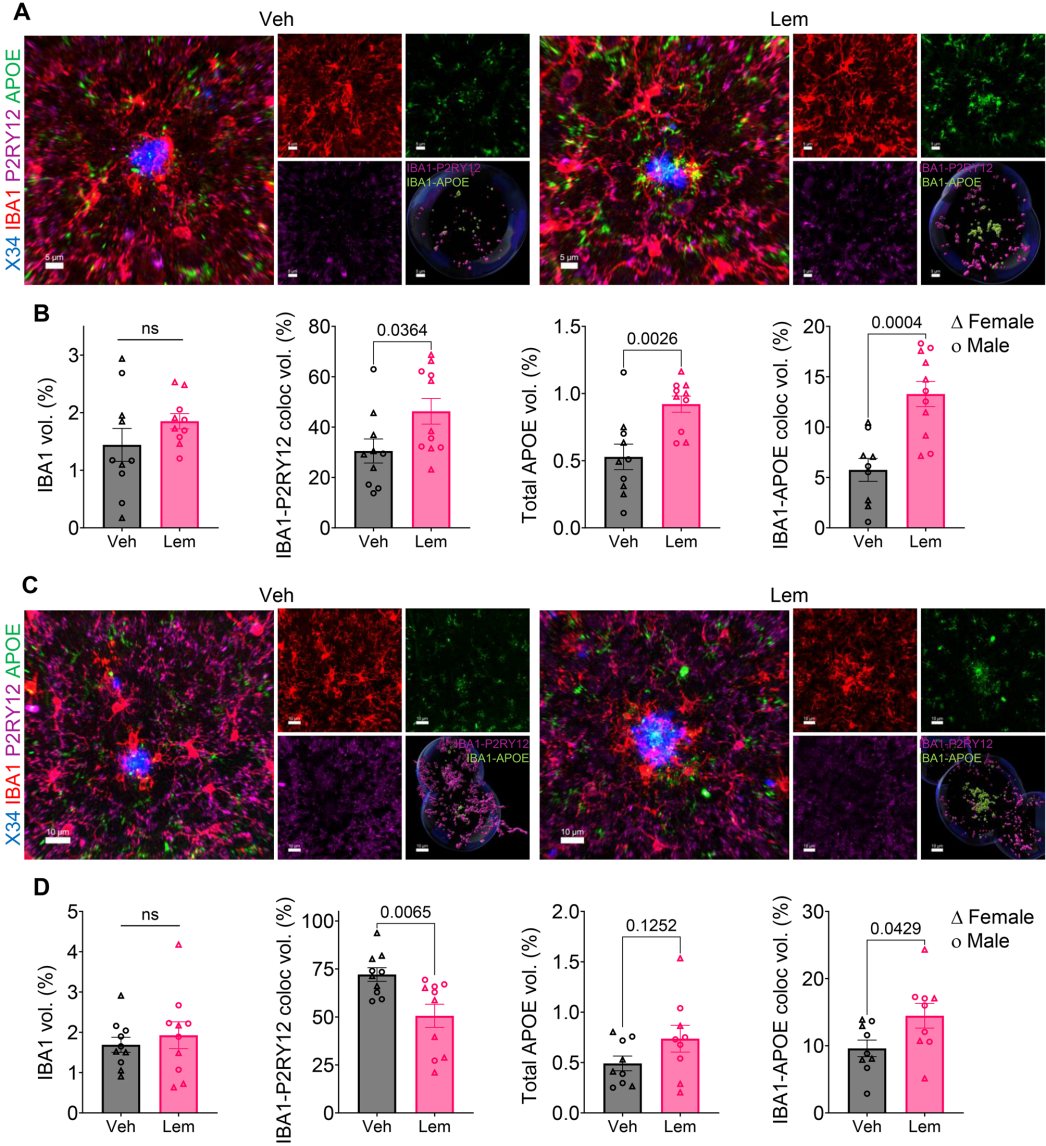
Immunofluorescent validation of lemborexant-induced microglial phenotype shifts. (**A–B**) Representative confocal images and quantification of peri-plaque microglial P2RY12 and APOE in PSAPP mice treated from 3/3.5 to 4.5/5 months of age (timeline in Fig. 1A). (**C–D**) Representative confocal images and quantification of peri-plaque microglial P2RY12 and APOE in PSAPP mice treated from 9 to 10 months of age (timeline in Fig. 3A). Data are mean ± SEM; exact *p* values shown in graphs

We acknowledge that treating individual cells as independent observations may inflate statistical power, particularly for genes without proportional differences. Nonetheless, key scRNA-seq findings were validated histologically: LEM increased *Cd68* transcript levels (Supp Fig. S8A) and peri-plaque CD68 protein expression (Figs. 2A,B and 3F,G). Age-dependent shifts in peri-plaque P2RY12 and APOE expression mirrored transcriptional changes in older mice (Fig. 4F; Supp Fig. S9). In younger mice (3/3.5–4.5/5 months; Fig. 1A), LEM modestly elevated P2RY12 and robustly increased APOE (Fig. 5A,B), whereas in older mice (9–10 months; Fig. 3A), LEM reduced P2RY12 while maintaining elevated APOE (Fig. 5C,D). Together, these data support a LEM-induced shift toward a DAM-like microglial phenotype.

### Microglial depletion abolishes lemborexant’s anti-amyloid effects

**Fig. 6:**
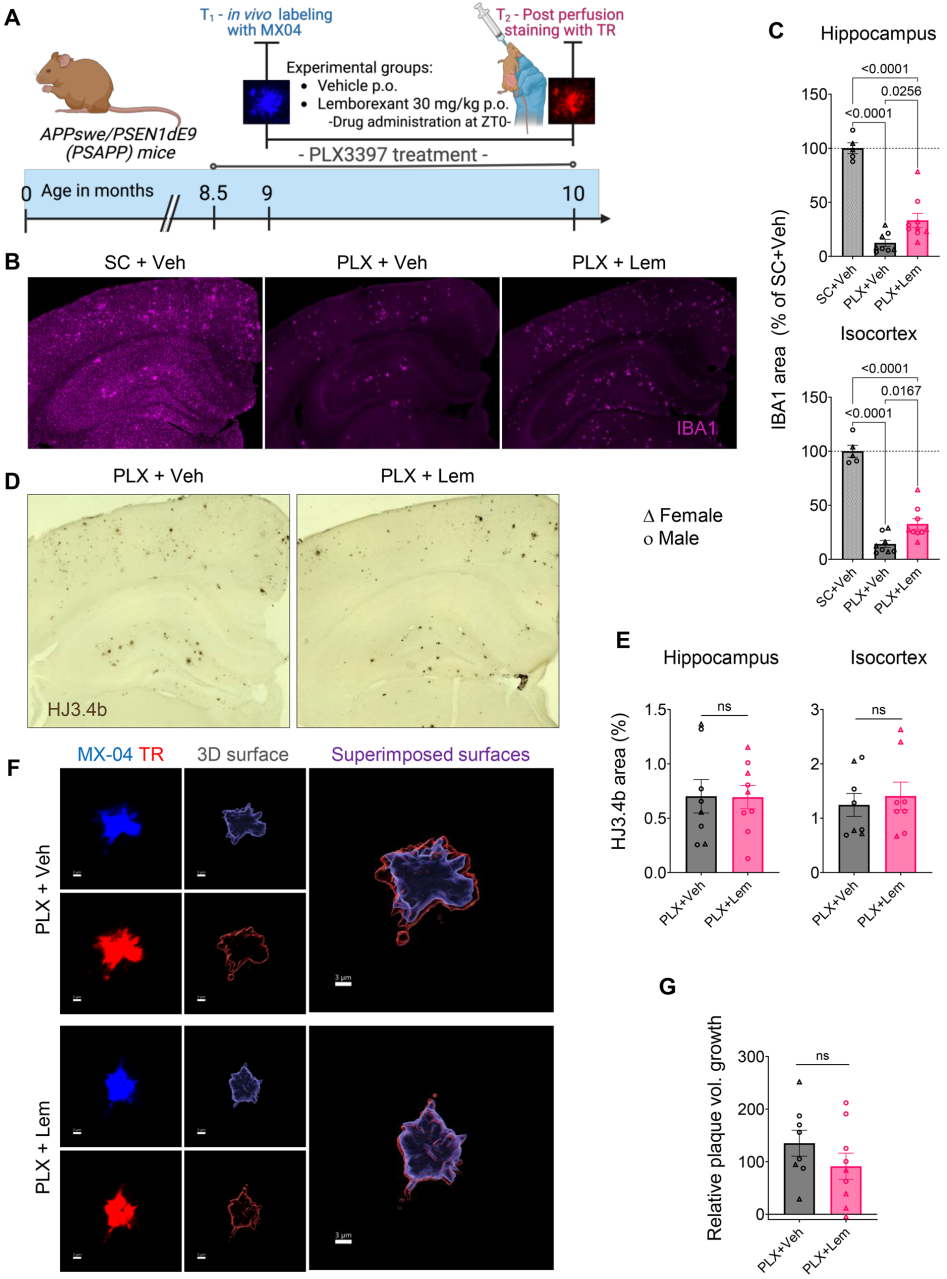
Microglial depletion abolishes the anti-amyloid effects of lemborexant. (**A**) Experimental timeline and dosing scheme. (**B–C**) Verification of microglial depletion: representative images and quantification of IBA1+ immunofluorescence after PLX3397 treatment. (**D–E**) Representative images and quantification of total plaque burden (HJ3.4b) in VEH and LEM groups under PLX3397. (**F–G**) Representative MX-04/thiazine red reconstructions and quantification of plaque core growth after microglial depletion. Data are mean ± SEM; exact *p* values shown in graphs

To determine whether microglia are required for LEM’s effects on plaque dynamics, we depleted microglia in 8.5-month-old PSAPP mice using the CSF1R antagonist PLX3397 [49, 50] and repeated our 30-day plaque growth assays. PLX3397 could not be used in younger mice, as it selectively inhibits early plaque formation [51, 52]. PLX3397 treatment began 14 days before MX-04 timestamping at 9 months, followed by daily VEH or LEM for one month (Fig. 6A). PLX3397 dramatically reduced IBA1+ microglial density, though a subset—particularly peri-plaque microglia—persisted, consistent with reports that fully activated/DAM-like microglia are less CSF1R-dependent for survival [53, 54]. The expanded proliferative microglial population observed after LEM treatment in our scRNA-seq data (Fig. 4B,C) may also confer survival advantage under PLX3397 (Fig. 6B,C).

At 10 months, plaques were labeled with thiazine red and growth measured by subtracting MX-04 from thiazine red volume. Total plaque burden by HJ3.4b IHC did not differ between VEH- and LEM-treated groups under microglial depletion (Fig. 6D,E), and LEM showed no significant effect on plaque growth, though a non-significant trend toward reduced growth was noted (Fig. 6F,G). These findings demonstrate that microglia are essential for LEM’s protective effects against amyloid pathology while suggesting additional contributing mechanisms.

## Discussion

Aβ accumulation is the earliest detectable hallmark of AD, emerging 15–20 years before clinical symptoms in both sporadic [55, 56] and autosomal dominant forms [57]. The rate of Aβ deposition reliably predicts cognitive decline [58], defining a critical window between biomarker detection and symptom onset. Recent trials demonstrate that early anti-Aβ interventions can decelerate cognitive and functional decline in mild cognitive impairment or early AD [59], and long-term gantenerumab therapy in presymptomatic individuals identified by amyloid PET achieved approximately 50% reduction in dementia progression [60]. Prevention of plaque formation or slowing of plaque growth in the preclinical phase is thus a promising therapeutic strategy.

Sleep is increasingly recognized as vital to brain homeostasis, with chronic disturbances contributing to AD pathogenesis [61]. In PSAPP mice, chronic sleep deprivation or fragmentation accelerates plaque growth [4, 11]. Sleep enhances glymphatic function, and sleep deprivation impairs clearance of amyloid PET tracer [62] or MRI contrast in healthy adults [63]. Even a single night of sleep deprivation can increase Aβ42 production and elevate CSF Aβ42 in healthy middle-aged adults [6, 64], and selectively disrupting slow-wave sleep is sufficient to raise CSF Aβ levels [65]. These findings implicate sleep quality as a modifiable risk factor for AD and suggest that restoring sleep architecture—particularly slow-wave sleep—may mitigate amyloid accumulation [66].

Central to sleep regulation are the orexin neuropeptides—orexin-A and orexin-B—derived from prepro-orexin [12] and acting through OX1R and OX2R [14]. Beyond their roles in feeding and energy homeostasis [14], orexins modulate reward-seeking [67], addiction [68], and sleep–wake regulation [13, 69]. The orexin system is conserved across rodents and humans, with OX1R preferentially binding orexin-A while OX2R binds both peptides with similar affinity [70]. In PSAPP mice, disruption of orexin signaling alters sleep–wake rhythms and Aβ dynamics, linking orexin signaling to sleep homeostasis and amyloid pathology [4, 71].

Genetic deletion of orexin mitigates amyloid plaque formation in AD mouse models [15], and acute suvorexant treatment lowers CSF Aβ42 in healthy humans [18], supporting orexin receptor antagonism as a therapeutic strategy. However, the efficacy of FDA-approved DORAs and mechanisms in AD models have remained unclear. Our findings address this gap: lemborexant (LEM) robustly suppressed plaque formation and slowed plaque growth in PSAPP mice, outperforming the non-DORA hypnotic doxepin (DOX) across multiple measures.

LEM increased sleep by both piezoelectric monitoring and polysomnography. However, the piezoelectric system overestimated sleep compared to EEG/EMG, particularly in DOX-treated mice. By EEG/EMG, LEM significantly increased total and non-REM sleep across the rest phase, whereas DOX increased total sleep only immediately after dosing and again mid-active phase without affecting REM or NREM—suggesting DOX promotes quiet wakefulness rather than deep sleep, consistent with its antihistaminic sedative profile. Because LEM produced greater physiological sleep enhancement than DOX, we cannot conclude that its stronger anti-amyloid effects reflect sleep-independent mechanisms. Rather, LEM’s benefits may stem from its ability to promote NREM sleep. Whether orexin signaling influences amyloid pathology independently of sleep remains to be determined, but our data support a model in which orexin receptor antagonism confers benefit at least partly through enhanced microglial phagocytic function.

Despite this limitation, prior studies suggest DORAs may modify AD-related pathology in ways that non-DORA agents do not. In P301S tauopathy mice, LEM prevented tau accumulation whereas zolpidem did not [72]. In humans, suvorexant reduced CSF Aβ while sodium oxybate failed to do so [6, 18]. These findings, together with ours, raise the possibility that orexin receptor antagonism exerts disease-modifying effects beyond generalized sleep promotion, though direct comparison at equivalent sleep enhancement will be needed to isolate sleep-independent mechanisms.

Long-term LEM administration initiated before plaque onset reduced both fibrillar and diffuse Aβ deposition in a dose-dependent manner, consistent with prior studies using almorexant in PSAPP mice [4]. By contrast, DORA-22 failed to reduce plaques in the aggressive 5XFAD model despite enhancing sleep [73], possibly due to rapid plaque kinetics or later treatment initiation. In mice with established pathology, LEM reduced diffuse plaque burden and slowed mature plaque growth by ~50% using MX04 timestamping.

The amyloid hypothesis has evolved to integrate neuroimmune interactions, with microglia emerging as critical modulators of plaque dynamics [74, 75]. Microglia continually survey the brain parenchyma and assume distinctive states that shift over time [27, 76]. Around amyloid plaques in both humans and AD mice [22, 24, 60], microglia display phenotypes that transition from early proliferative/phagocytic states to later pro-inflammatory states, suggesting that directed phenotypic modulation may alter disease trajectories [44, 77–81]. In PSAPP mice, peri-plaque microglia proliferate and upregulate CD68 without broad inflammatory gene induction [42], features mirrored in APP23 mice [40] that likely reflect an early protective response. We found that LEM amplified this protective microgliosis, increasing peri-plaque CD68 expression and Aβ uptake in vivo (Fig. 2). Notably, chronic optogenetic stimulation of cortical GABAergic interneurons also enhances sleep and reduces plaques by driving microglial phagocytosis in PSAPP mice [82], further linking sleep regulation to microglial function. LEM’s effect on CD68 was replicated when treatment began after moderate plaque accumulation (Fig. 3), and single-cell transcriptomics revealed an expanded proliferative microglial population (Fig. 4).

Single-cell analysis showed that although terminal DAM and IFN-induced clusters remained largely unchanged, LEM induced a broader transition toward DAM-like states through modest but consistent marker upregulation within LEM-enriched clusters. Differential analysis confirmed increased fractions of cells expressing DAM markers (*Apoe*, *Cd63*, *Gpr84, Itgax*) and decreased fractions expressing homeostatic genes (*C5ar2*, *Gp9*, *Slc1a3*). Additional DAM-associated genes (*Cd68*, *Ctsz*, *Axl*, *B2m*, *Ctsd*, *Lyz2*, *Tyrobp*) increased in expression without proportional changes, while homeostatic transcripts (*Cst3*, *Jun*, *P2ry12*) decreased—consistent with a shift from resting to activated states [44]. Given that PSAPP mice accumulate plaques more gradually than aggressive models [20, 44, 83], these shifts are expectedly subtle. LEM also expanded a CD11c^+^ subset (*Itgax*, *Colec12*) linked to developmental myelination and lysosome-rich phagocytic activity [46–48]. Several phagocytosis-related genes increased in both expression and cell fraction (Fig. 4E; Supp Fig. S8), notably Hcar2—the niacin receptor critical for microglial Aβ clearance [84]—and Fgl2, whose plasma levels inversely correlate with amyloid accumulation [85]. Interferon-inducible genes (*Ifi27l2a, Ifitm3, Ly6e*) similarly increased, whereas MHC-I components (*H2–D1*, *H2–K1*) rose only in expression level (Fig. 4; Supp Fig. S8). Cytokine and chemokine transcripts remained stable, maintaining a non-inflammatory profile (Supp Table T1). Selective MHC-I upregulation suggests possible engagement of adaptive immunity, potentially involving CD8+ T cells, though this remains speculative and warrants future investigation [86].

Recent work indicates microglia actively influence plaque dynamics throughout disease, with distinct phenotypes contributing differently to progression. Notably, homeostatic microglia appear essential for plaque initiation [52]. Our findings show LEM elicits stage-specific responses: in young mice, microglia adopted a transitional state with high co-expression of P2RY12 and APOE, correlating with reduced plaque initiation; in aged mice, LEM promoted a greater phenotypic shift marked by decreased P2RY12 and sustained APOE expression (Fig. 5).

To test whether microglia are required for LEM’s effects, we employed PLX3397-mediated depletion. Microglial removal abrogated LEM’s capacity to slow plaque growth (Fig. 6), confirming microglia as essential mediators, though a non-significant trend toward reduced growth suggests additional microglia-independent mechanisms.

The mechanisms underlying LEM-induced microglial changes remain unclear. Although orexin is not widely recognized as a neuroinflammation regulator, sparse literature suggests interactions between orexin signaling and microglial function. Microglia upregulate OX1R following LPS treatment in vitro and stroke in vivo [87], and orexin treatment in BV2 cells can inhibit Aβ phagocytosis and impair autophagic flux [88]. However, in our BV2 experiments, neither orexin-A nor LEM directly affected phagocytosis, supporting the hypothesis that LEM’s microglial effects are indirect—potentially through improved sleep [82], or interactions with other cell types, particularly neurons. Cell-type-specific deletion of OX1R and OX2R in AD models will be needed to address this definitively.

From a translational perspective, DORAs offer practical advantages over traditional sedative-hypnotics: they produce less next-day sedation, fewer falls, and reduced cognitive impairment compared to benzodiazepines and antihistamines [89]. DOX, while effective at increasing piezoelectric-measured sleep duration, induced sedation during the active phase—a profile that may limit its utility in older adults at risk for falls. LEM’s selective enhancement of true sleep without active-phase sedation positions DORAs as attractive candidates for long-term use in early AD. This is particularly relevant given that AD can be diagnosed at preclinical or early symptomatic stages based on amyloid biomarker positivity, and emerging evidence suggests amyloid accumulation beyond a critical threshold precipitates cognitive decline [56]. DORAs may therefore be most beneficial in individuals with early amyloid pathology, where slowing accumulation over years could delay progression toward this threshold.

In summary, LEM exerts potent anti-amyloid effects by augmenting microglial phagocytic and DAM-like phenotypes without inciting overt neuroinflammation. These data support the concept that immunomodulation, rather than broad immunosuppression, may offer an effective therapeutic approach for AD [90], and position DORAs as promising therapeutics that leverage sleep regulation and neuroimmune interplay.

## Conclusion

Our findings demonstrate that lemborexant, an FDA-approved DORA, prevents amyloid plaque formation in young PSAPP mice and slows plaque growth in mice with established pathology—effects that exceed those of the comparator sleep medication doxepin. Although both drugs increased total sleep by piezoelectric monitoring, only lemborexant enhanced NREM sleep by EEG, prevented fibrillar plaque accumulation, and slowed growth of existing plaques. Mechanistically, lemborexant amplified microglial CD68 expression, enhanced Aβ phagocytosis, and promoted a transcriptional shift toward an activated, DAM-like phenotype without inducing overt inflammation. Microglial depletion abolished these benefits, establishing microglia as essential mediators. Because lemborexant also increased NREM sleep more effectively than DOX, its microglial and anti-amyloid effects may be downstream of improved sleep quality. These findings position DORAs as promising therapeutics for early AD, uniquely coupling physiological sleep promotion with beneficial microglial modulation.

## Electronic supplementary material

Below is the link to the electronic supplementary material.

Supplementary Material 1

Supplementary Material 2

## Data Availability

The scRNAseq dataset is freely available on the NIH GEO website under accession number GSE312840.
