# Supplementary material for: Dual orexin receptor antagonism with lemborexant enhances microglial clearance of β-amyloid in mice
Source: Mol Neurodegener. 2026 May 22;21:28. doi: 10.1186/s13024-026-00948-y (PMC13214429; doi:10.1186/s13024-026-00948-y)
Supplement: Supplementary file 1 — Supplementary Material 1 [file 13024_2026_948_MOESM1_ESM.pdf]

Supplementary Figures & Table

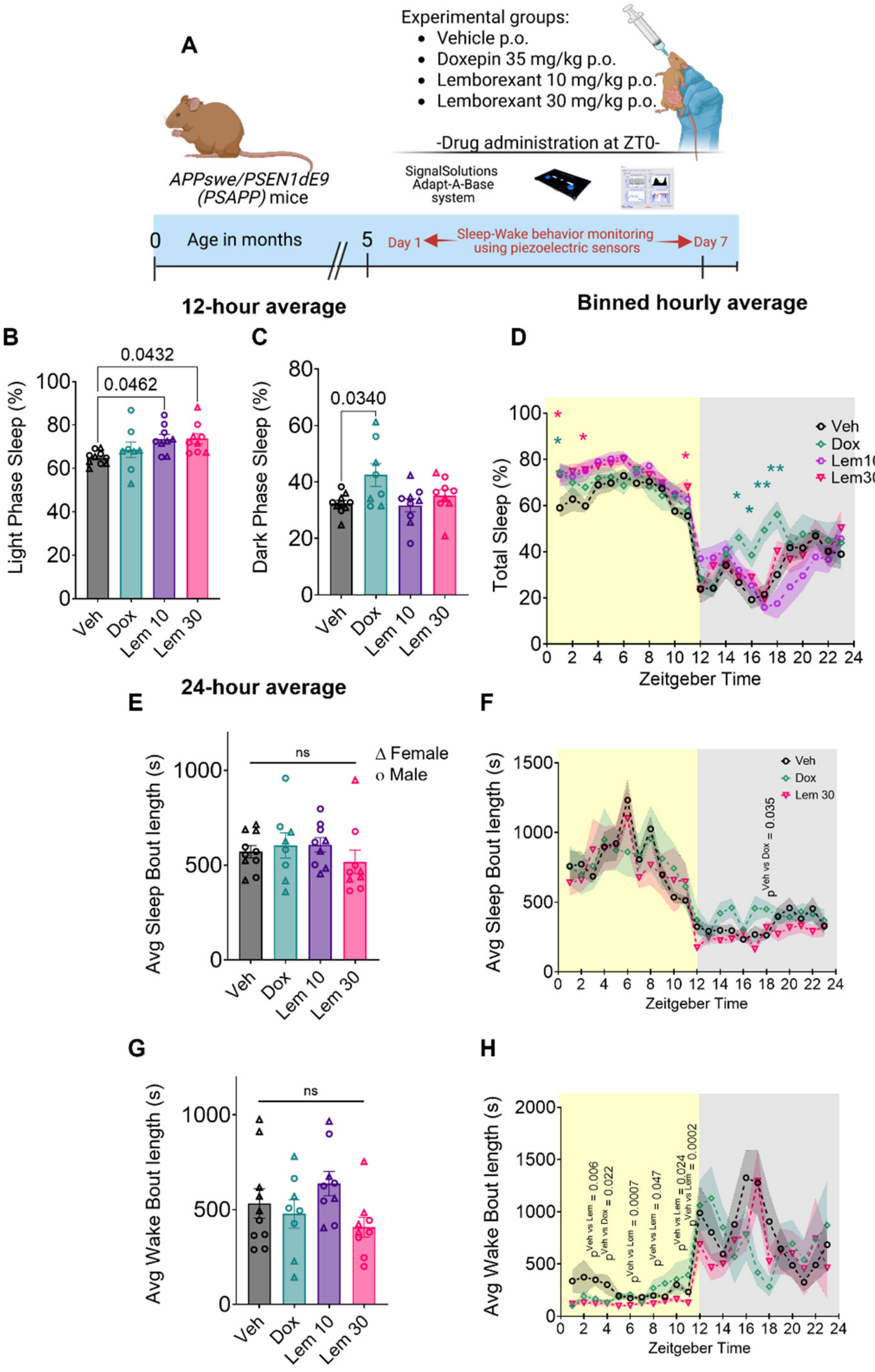

Supplementary Figure S1. Additional Sleep/wake metrics. (A) Sleep recording timeline and dosing scheme. (B–C) Phase-specific average percent sleep. (D) Circadian hourly binning of percent sleep for VEH, DOX, LEM10, and LEM30. (E-H) Average sleep and wake bout lengths (E,G), and circadian binning (F,H) (mean ± SEM; significant P values indicated). Each circle indicates one male mouse, each triangle one female.

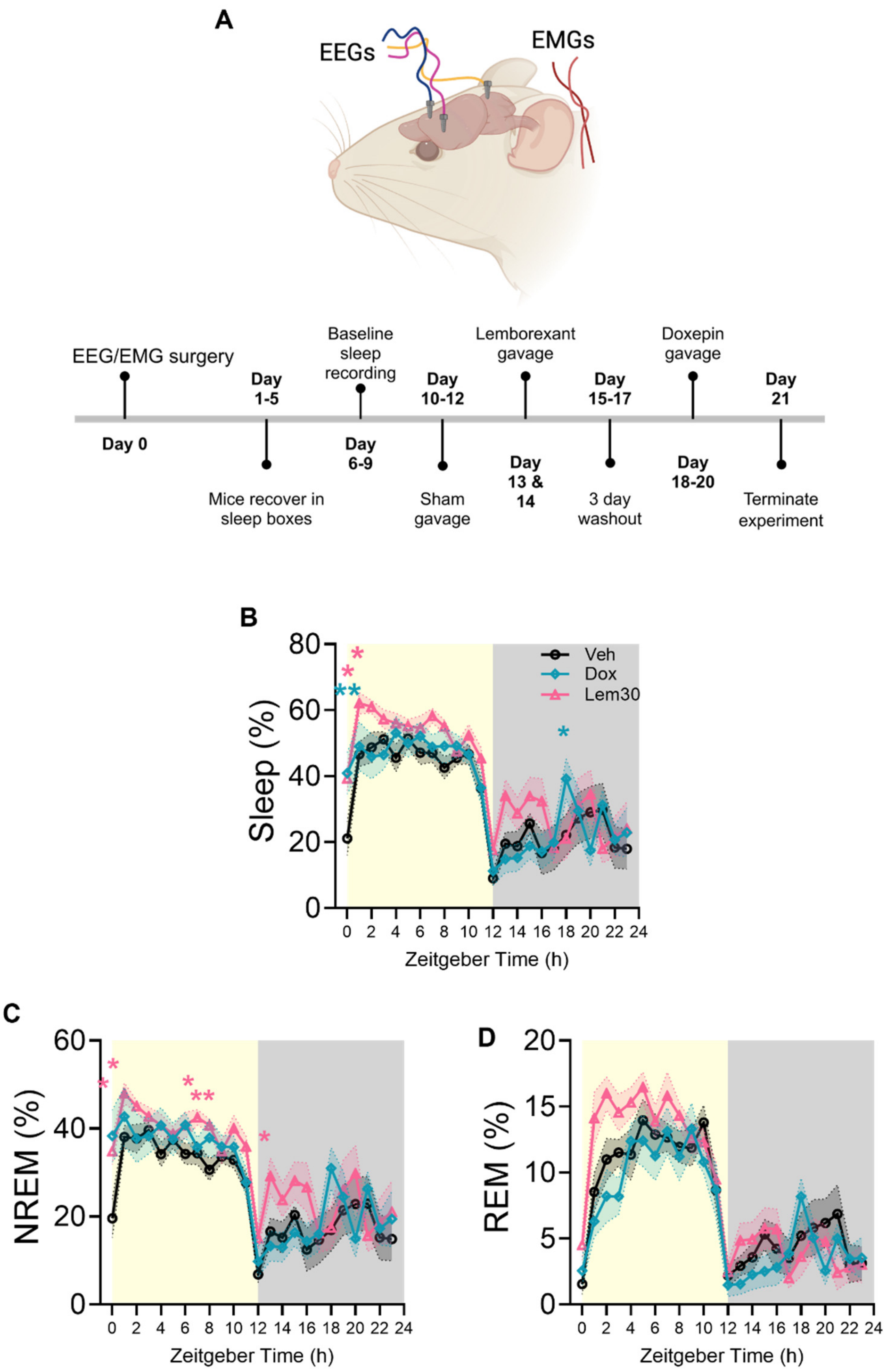

Supplementary Figure S2. EEG/EMG validation Sleep/wake metrics. (A) Stereotactic surgery, dosing and sleep recording timeline. (B–D) Circadian hourly binning of percent total sleep (B), non-REM sleep (C), and REM sleep (C). (mean ± SEM; 2-Way ANOVA with Tukey's multiple comparison test, *P < 0.05, **P < 0.01 compared to VEH).

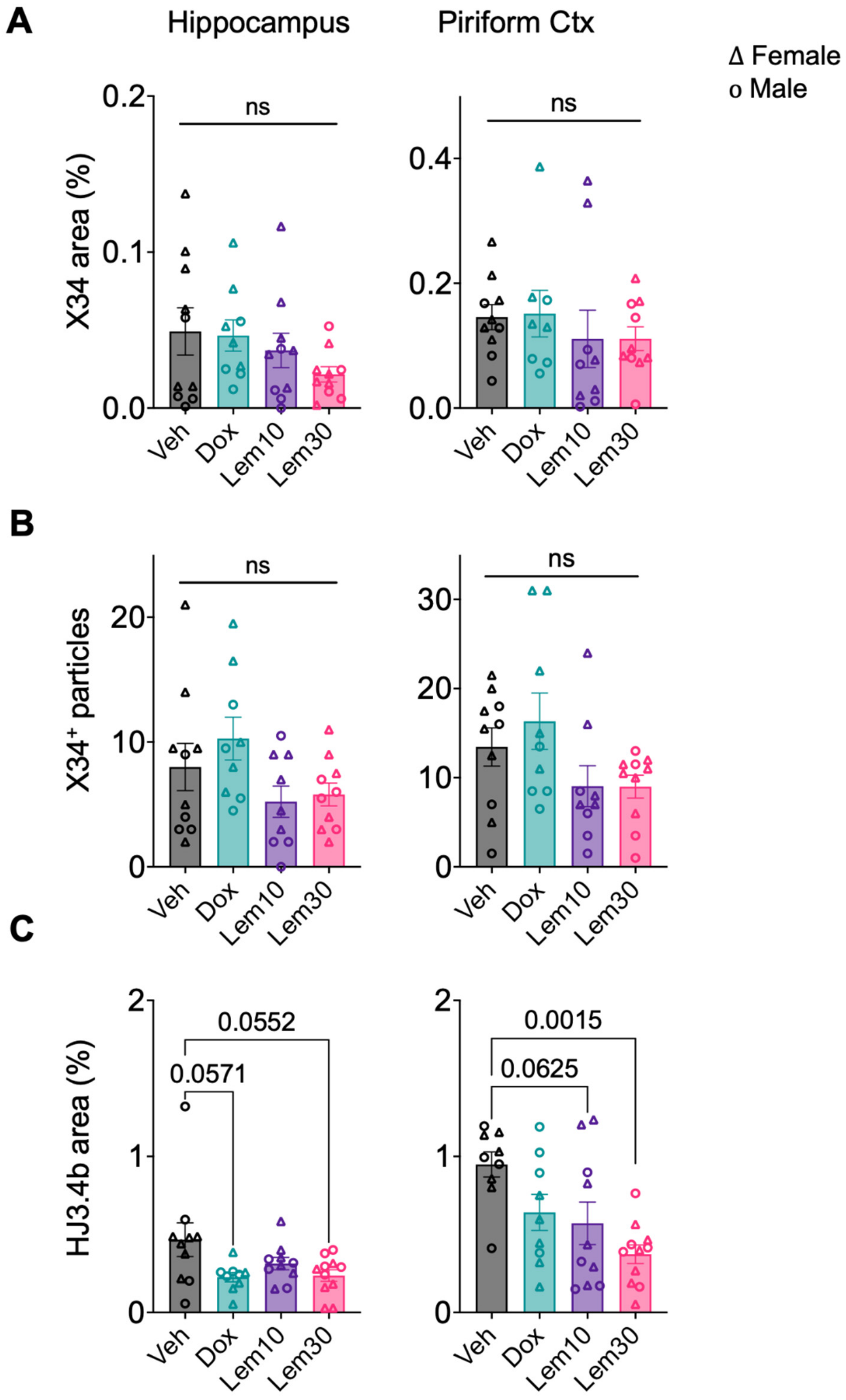

Supplementary Figure S3. Regional plaque analysis. Plaque burden in hippocampus and piriform cortex, by X34 % area (A), X34+ particles (B), and HJ3.4b % area (C). Mean ± SEM is shown; significant P values indicated. Additional data from experiments in main Figure 2.

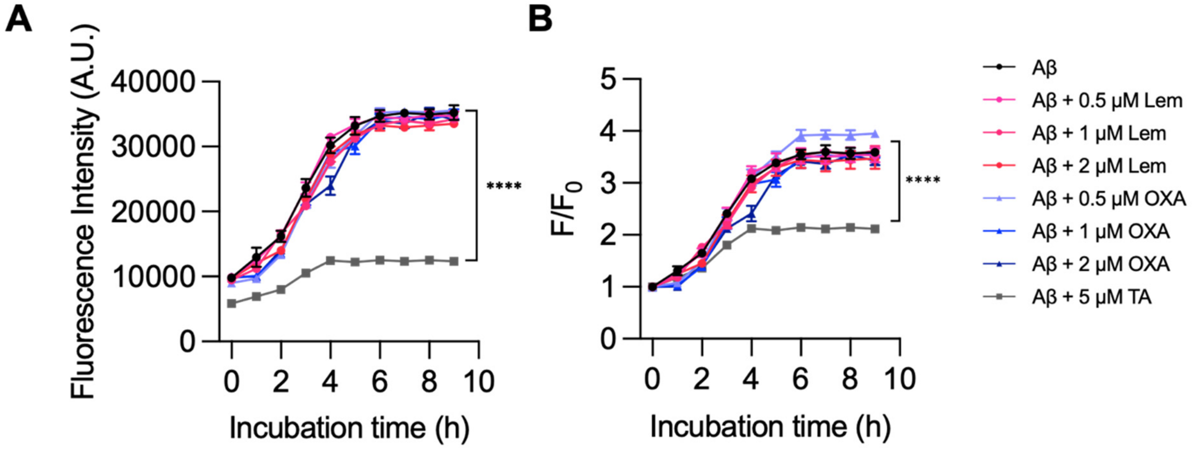

Supplementary Figure S4. Cell free amyloid aggregation assay. (A) Fluorescence intensity curve with thioflavin T (ThT) incubated with Aβ with or without varying concentrations of LEM or orexin-A (OXA). ThT-treated Aβ with 5 μM of tannic acid (TA) was used as a positive control to inhibit Aβ aggregation. Each dot represents the mean intensity from independent technical triplicates at each time point (Mean ± SEM, two-way ANOVA with Dunnett's multiple comparison, ****$P < 0.001$). (B) ThT fluorescence reported as fold change over baseline fluorescence intensity at 0h time point (F/F_0).

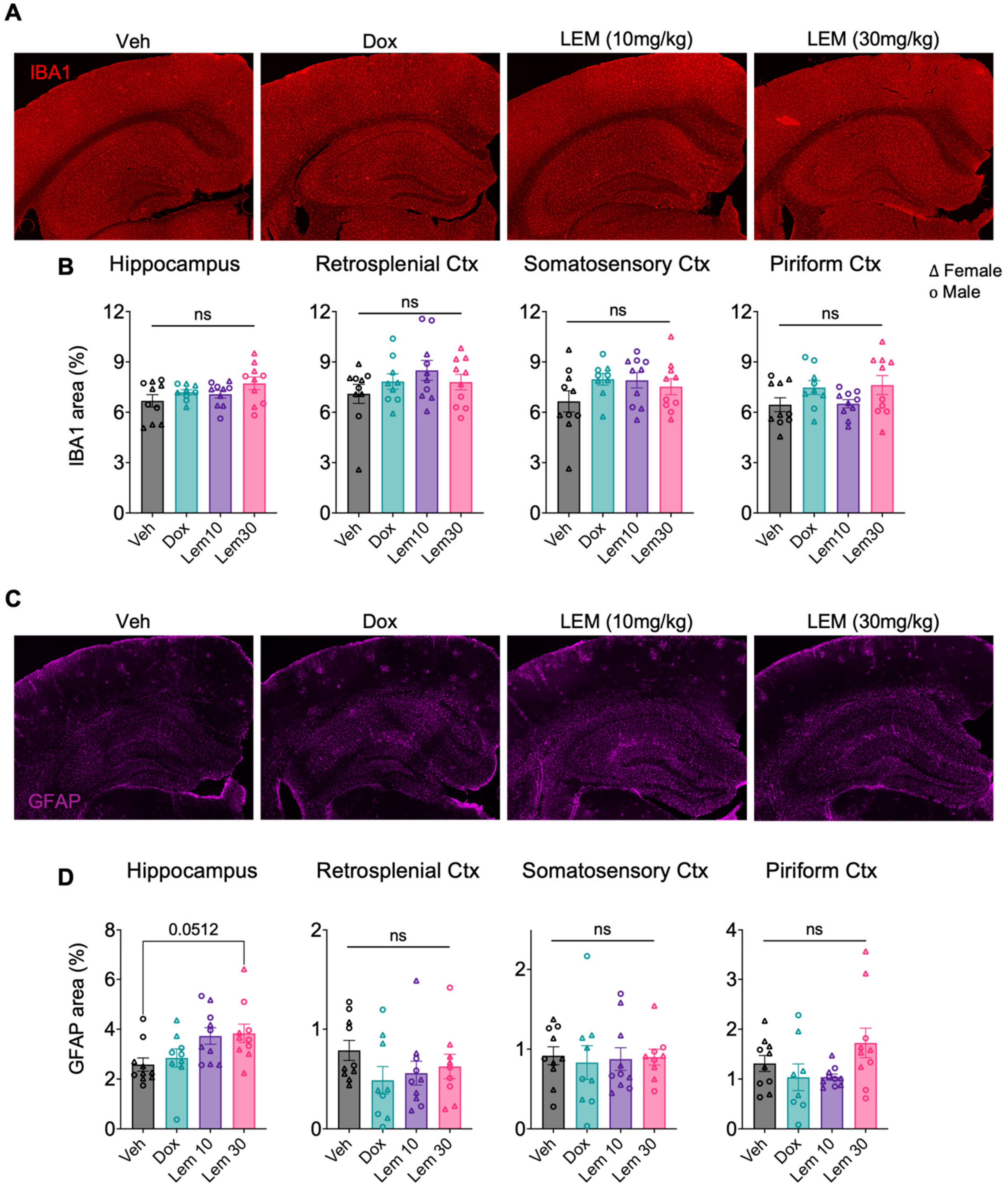

Supplementary Figure S5. Glial activation analysis. PSAPP mice were treated with veh, LEM, or DOX as in main Figure 2. (A–B) IBA1 immunoreactivity (% area) across four brain regions; no significant treatment differences were observed. (C–D) GFAP immunoreactivity across four brain regions; no significant treatment differences were observed. In all panels, mean ± SEM is shown, P values are listed if P<0.1, other n.s. (not significant) and are from one-way ANOVA.

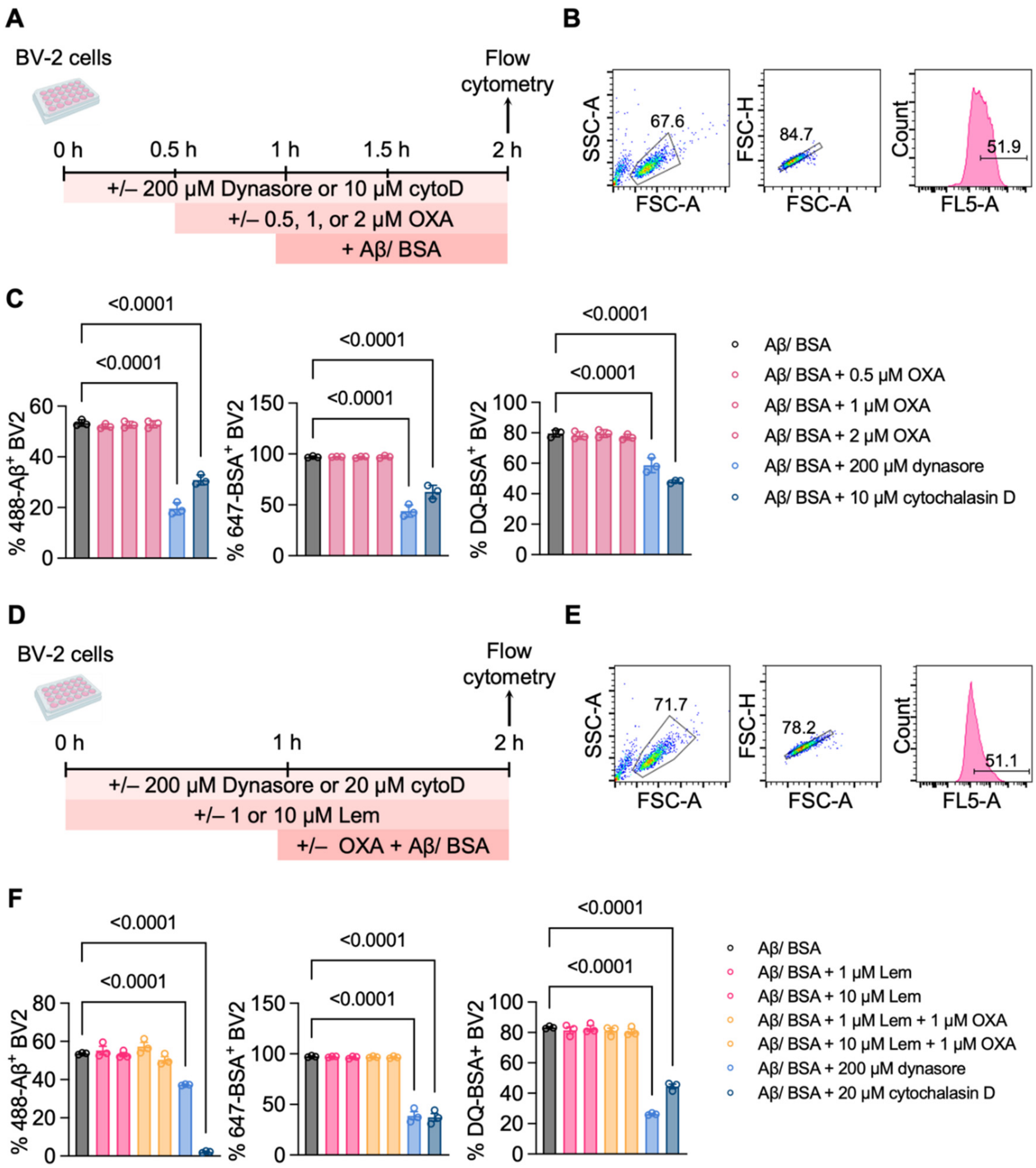

Supplementary Figure S6. In-vitro 488-Aβ, 647-BSA uptake and DQ-BSA degradation assay in BV-2 cells. (A) Schematic illustration of experiment timeline for 488-Aβ, 647-BSA uptake and DQ-BSA degradation assay using orexin-A (OXA)-treated BV2 cells. Dynasore and cytochalasin D (cytoD) were used as inhibitors of protein uptake. (B) Representative flow cytometry gating strategy used for analysis. (C) Quantification of the percentage of BV2 cells positive for 488-Aβ, 647-BSA, or DQ-BSA (Mean ± SEM; P value shown). (D) Schematic illustration of experiment timeline for 488-Aβ, 647-BSA uptake and DQ-BSA degradation assay using LEM and/or OXA-treated BV2 cells. Dynasore and cytoD were used as inhibitors of protein uptake. (E) Representative gating strategy. (F) Quantification of percentage of BV2 cells positive for 488-Aβ, 647-BSA, or DQ-BSA, indicating protein uptake and degradation (Mean ± SEM; P values shown for statistically significant difference only).

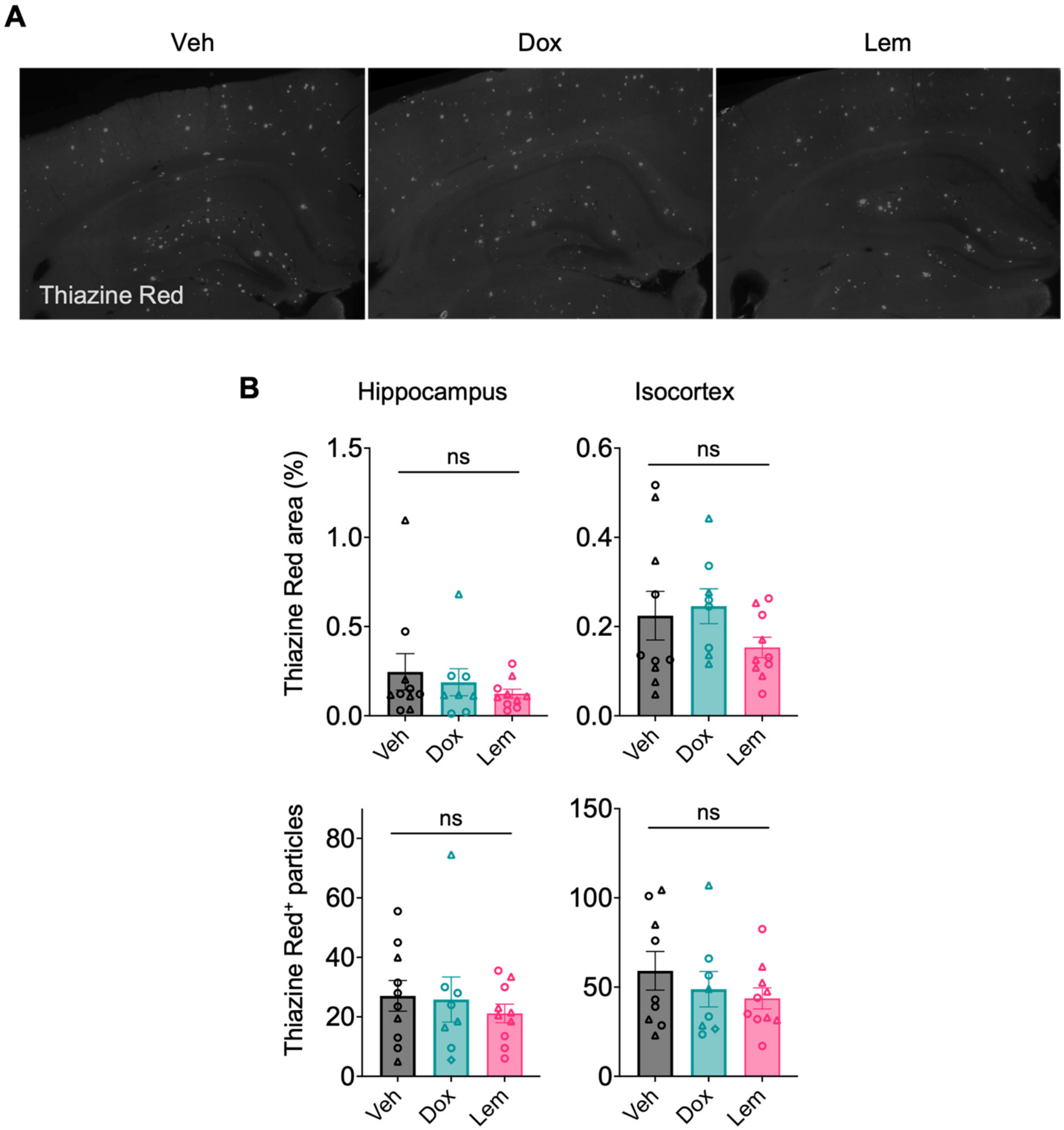

Supplementary Figure S7. Dense-core plaque analysis with thiazine red. Aged PSAPP mice were treated with VEH, DOX, or LEM as in main Figure 3A. Representative images of thiazine red staining (A) and quantification in hippocampus and isocortex (B) are shown. For all panels, mean ± SEM is shown, n.s. (not significant) for all analyses with one-way ANOVA.

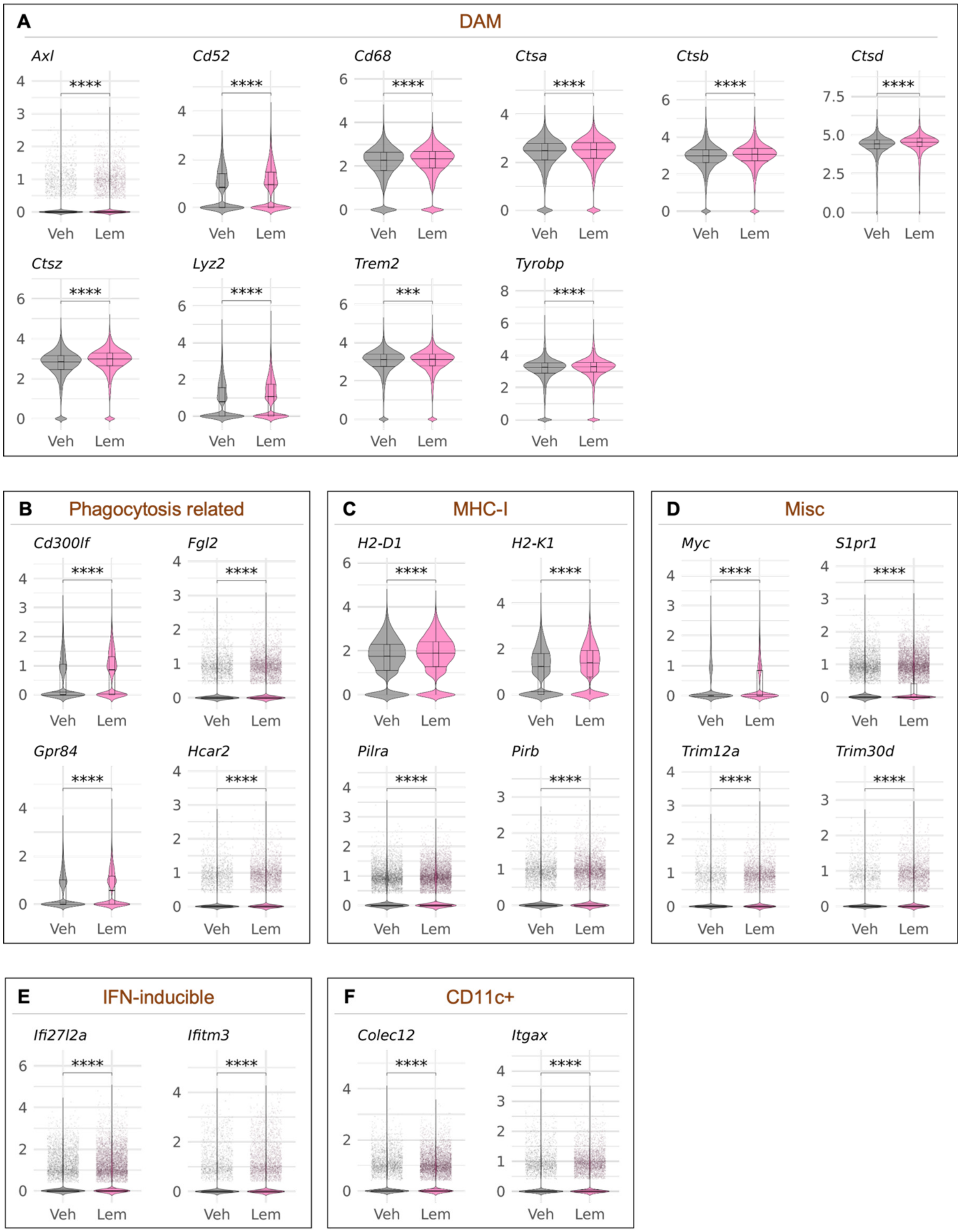

Supplementary Figure S8. Differential gene expression of selected upregulated genes in isolated microglia. Single cell RNAseq was performed on microglia isolated from veh or LEM-treated PSAPP mice, as described in main Figure 4. Violin plots of select upregulated genes after LEM treatment grouped by their molecular functions. ***P < 0.001, ****P < 0.0005 by Wilcoxon rank-sum test with FDR correction.

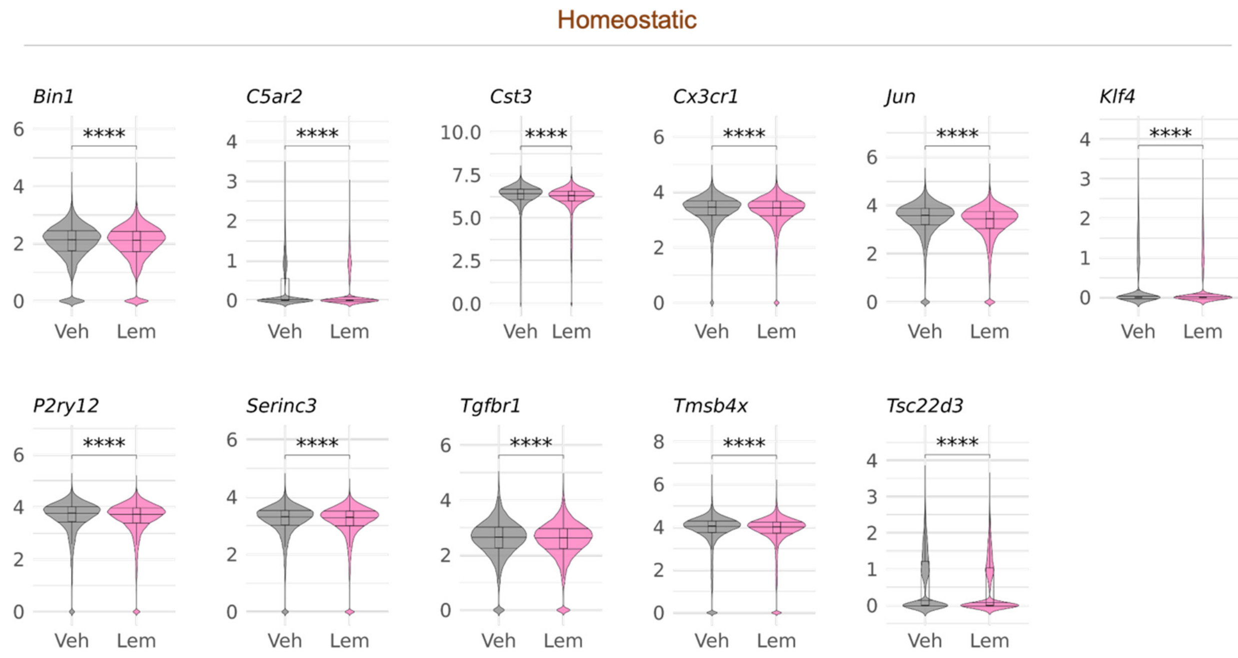

Supplementary Figure S9. Differential gene expression of selected downregulated genes. Single cell RNAseq was performed on microglia isolated from VEH or LEM-treated PSAPP mice, as described in main Figure 4. Violin plots of downregulated homeostatic genes after LEM treatment (****P < 0.0005 by Wilcoxon rank-sum test with FDR correction).

Gene	Direction of significant change
**Interleukins & TNF super-family**	
Il1a	Up
Il1b	Up
Il6	Up
Il10	No significant change
Il12b	No significant change
Il15	Down
Il18	Up
Il23a	No significant change
Tnf	Down
Tnfsf10	No significant change
Tnfsf6	No significant change
**Chemokines**	
Ccl2	No significant change
Ccl3	No significant change
Ccl4	No significant change
Ccl5	Up
Ccl7	No significant change
Ccl12	Down
Cxcl1	No significant change
Cxcl2	No significant change
Cxcl10	No significant change
Cxcl12	No significant change
**Colony-stimulating / growth factors**	
Csf1	No significant change
Csf2	No significant change
Csf3	No significant change

Supplementary Table T1. Cytokine and chemokine transcript changes. Changes in log average expression values for inflammatory genes across treatments using wilcoxon rank-sum test with FDR correction, based on scRNAseq data presented in main Figure 4.
