# Supplementary material for: Dual orexin receptor antagonism with lemborexant enhances microglial clearance of β-amyloid in mice
Source: Mol Neurodegener. 2026 May 22;21:28. doi: 10.1186/s13024-026-00948-y (PMC13214429; doi:10.1186/s13024-026-00948-y)

Original Western Blots: Sharma et al.

Cropped blots:

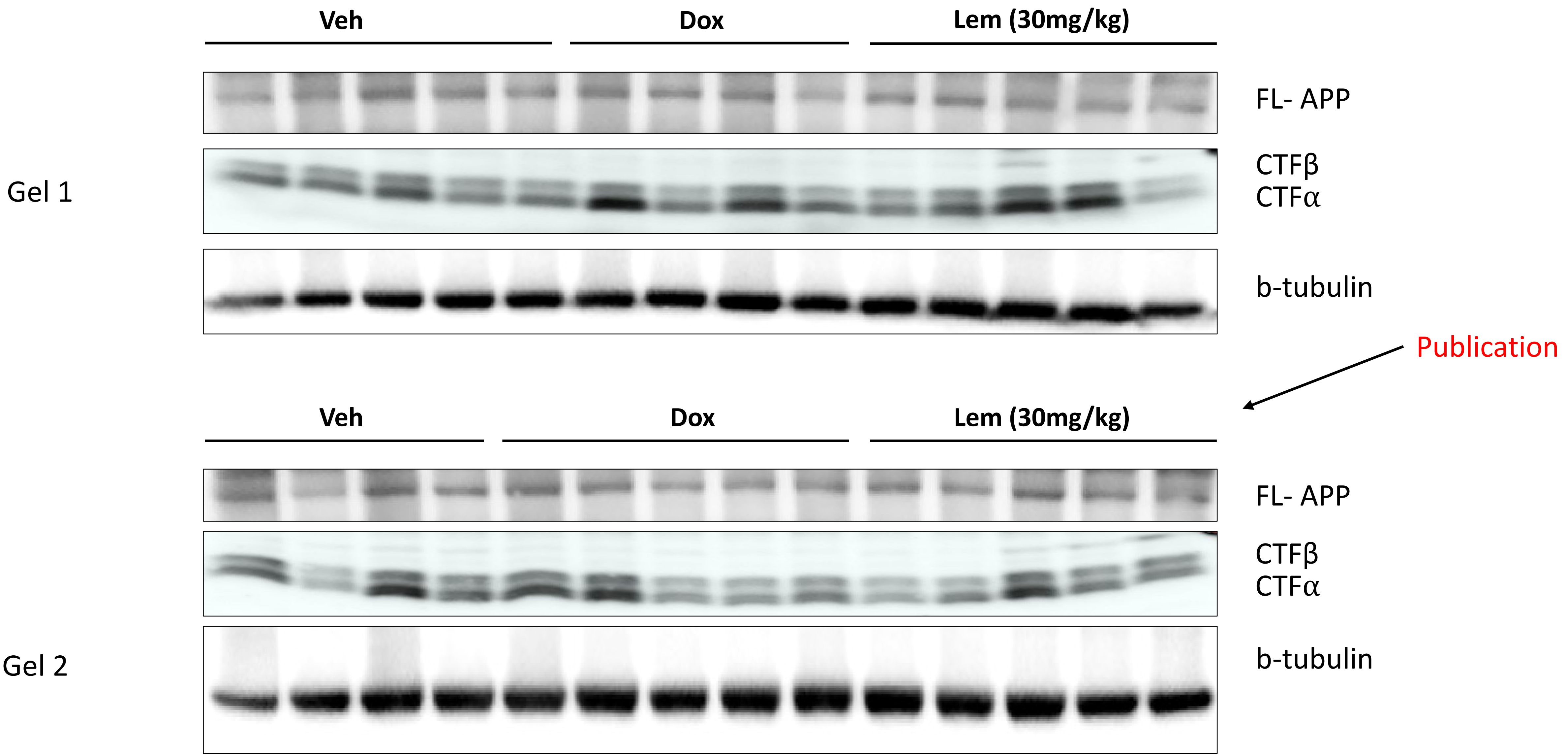

Full length APP (higher exposure)

Chemiluminescence

Gel1

Gel2

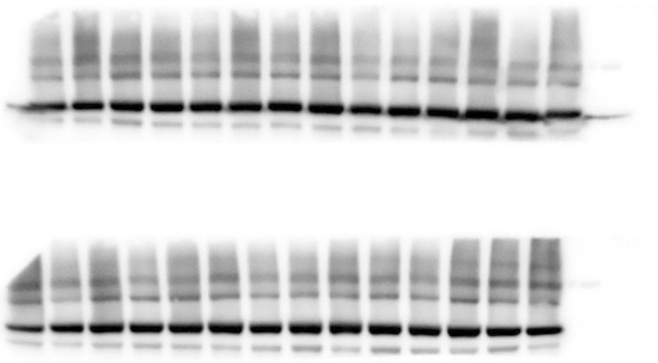

Composite

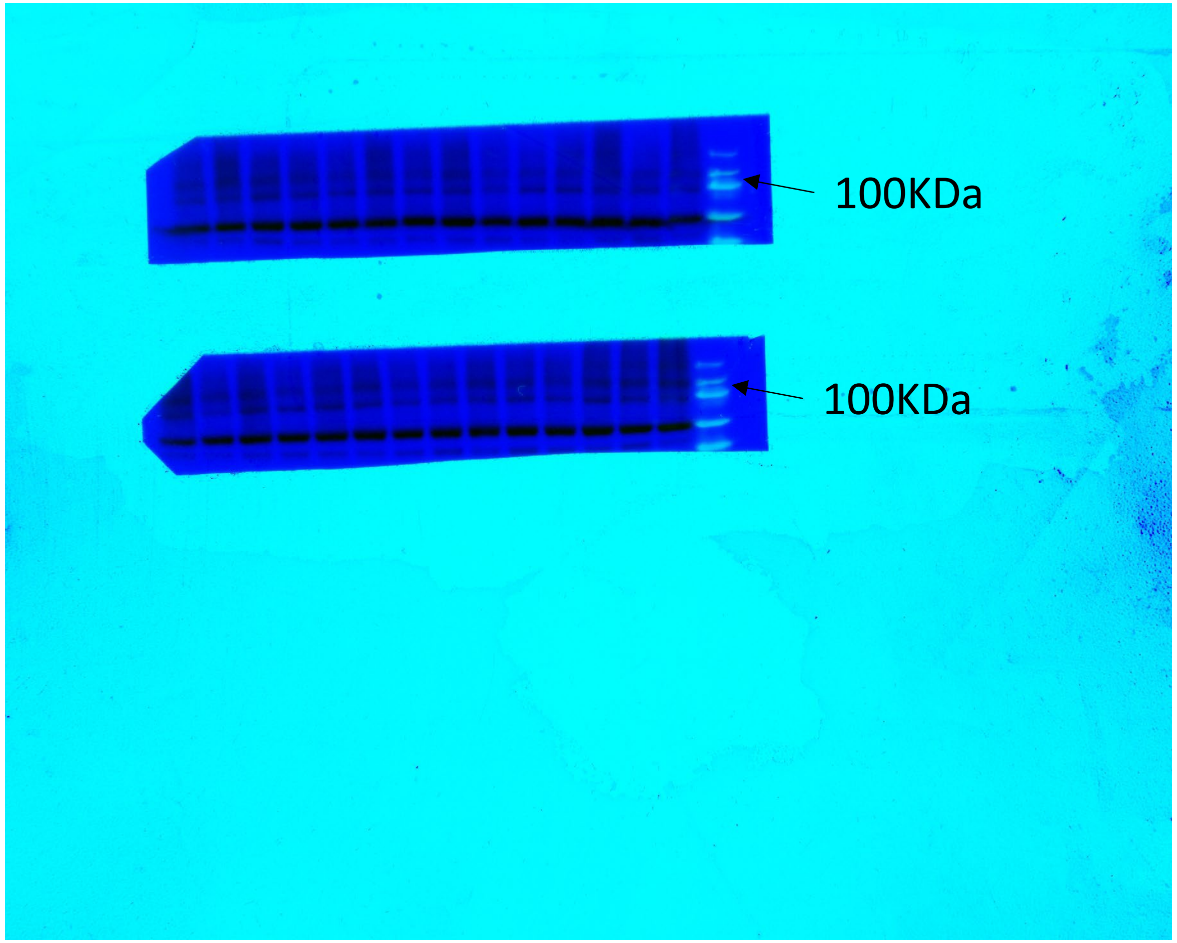

B-tubulin (lower exposure)

Chemiluminescence

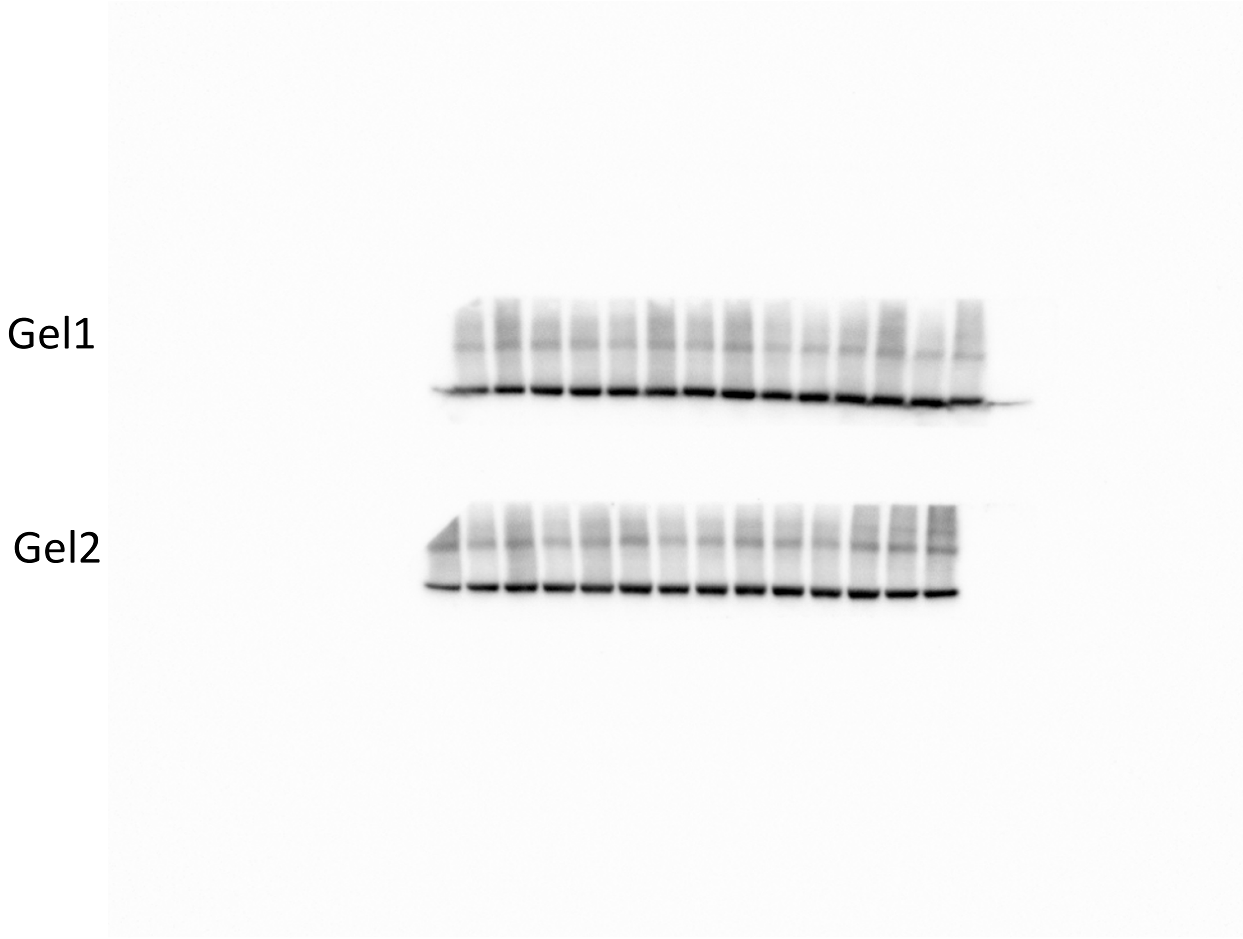

Composite

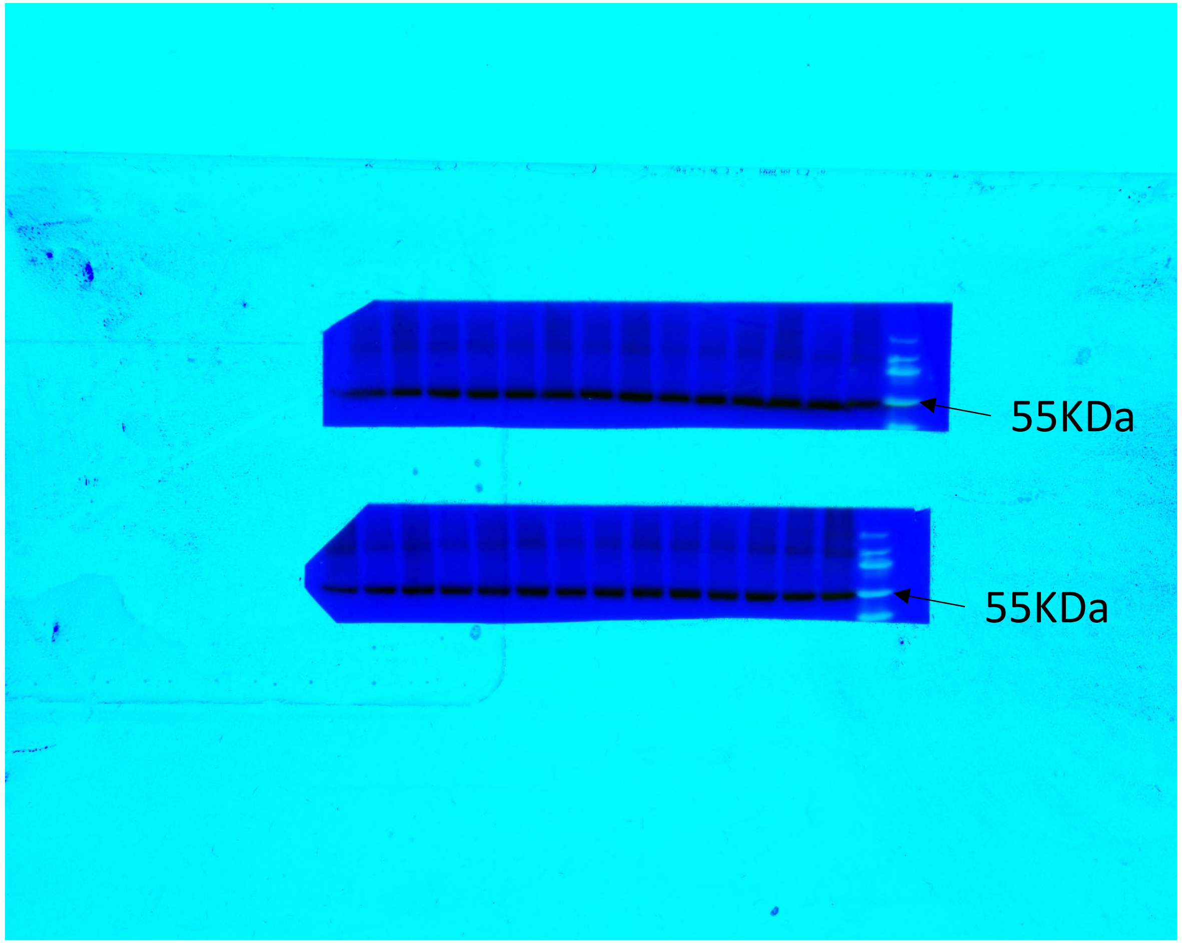

C-Terminal Fragments α and β

Chemiluminescence

Composite

Gel1

Gel2

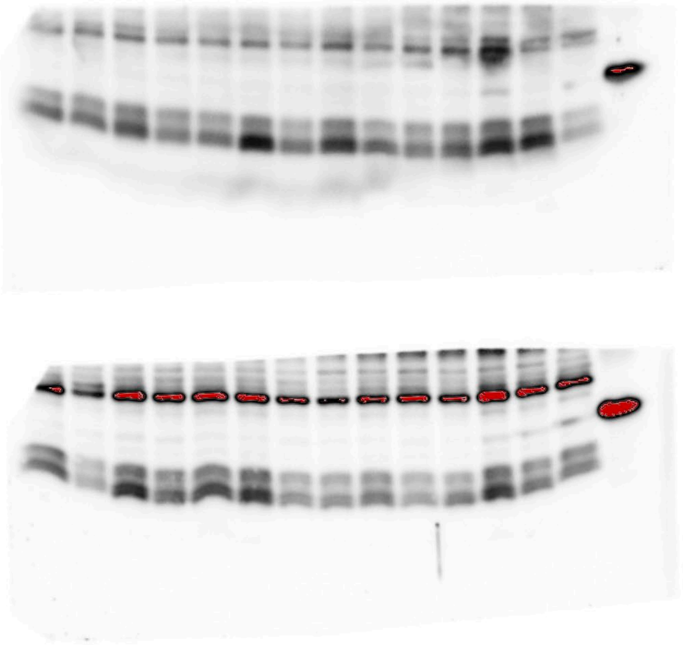

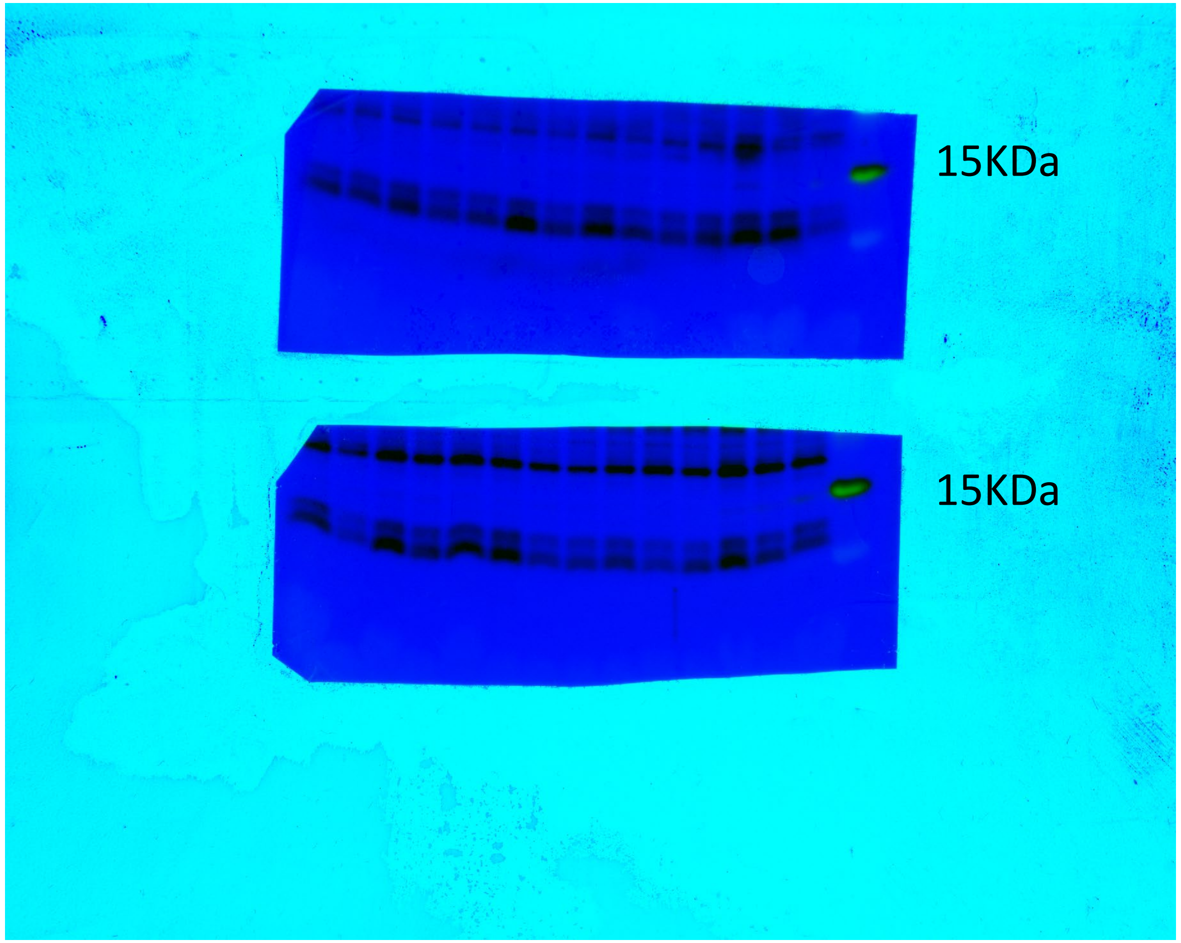

Supplement: Supplementary file 2 — Supplementary Material 2 [file 13024_2026_948_MOESM2_ESM.pdf]
